# Momentary anxiety and autonomic responses during everyday social interactions among patients with depression

**DOI:** 10.1038/s41398-026-03990-y

**Published:** 2026-04-04

**Authors:** M. Weiß, J. Gutzeit, A. Jachnik, E. C. Lampe, F. Rothbauer, M. Gründahl, S. Unterecker, S. Kittel-Schneider, G. Hein

**Affiliations:** 1 Department of Psychology I: Clinical Psychology and Psychotherapy, Institute of Psychology, University of Würzburg, Würzburg, Germany; 2 University Hospital Würzburg, Center of Mental Health, Department of Psychiatry, Psychosomatics and Psychotherapy, Translational Social Neuroscience Unit, Würzburg, Germany; 3 Department of Psychiatry, Psychosomatics and Psychotherapy, Sozialstiftung Bamberg, Bamberg, Germany; 4 Department of Psychiatry and Neurobehavioural Science, University College Cork, Cork, Ireland; 5 APC Microbiome Ireland, University College Cork, Cork, Ireland

**Keywords:** Human behaviour, Physiology

## Abstract

Social interactions play a central role in regulating affect and physiological arousal, with familiar and supportive relationships often associated with reduced anxiety and adaptive autonomic responses. However, it remains unclear whether individuals with depression benefit similarly from these so-called social buffering effects in everyday life. The present study examined momentary anxiety and cardiovascular responses (heart rate [HR] and heart rate variability [HRV]) during real-life social interactions in patients suffering from depression (*N* = 57) and matched healthy controls (*N* = 57). Participants reported on the familiarity and the gender of social interaction partners and the interaction context across five days, while a subsample also wore ambulatory electrocardiogram sensors. Across the patient and control group, higher interaction partner familiarity was associated with reduced state anxiety. However, only controls reported lower social interaction anxiety with increasing familiarity, whereas this pattern was not observed in patients. State social interaction anxiety was higher in controls when they interacted with female interaction partners, while there were no differences for patients. Regarding autonomic responses, patients exhibited higher baseline and state HR and lower baseline and state HRV compared to controls, consistent with autonomic dysregulation. We also observed lower HRs in opposite-gender interactions among controls but not among patients. Overall, these findings suggest that familiar social interactions are associated with lower levels of general state anxiety in both patients with depression and healthy controls, whereas social interaction anxiety and autonomic responses appear to show weaker associations with familiarity or the gender of interaction partners in patients. This research offers insights into how everyday social environments might support emotion regulation in clinical populations.

## Introduction

Human life unfolds against the backdrop of social contexts - interactions with friends, family, colleagues, and even strangers that shape the trajectory of daily experience [1, 2]. A large body of research underscores the ability of social connections to foster emotional well-being, revealing that supportive interactions are often linked to reductions in stress and anxiety, a phenomenon termed *social buffering* [3, 4]. In laboratory settings, for instance, participants who perform stressful tasks alongside a supportive partner report lower subjective anxiety and exhibit adaptive physiological responses, such as decreased heart rate (HR) and increased heart rate variability HRV; [5], but see also [6, 7]. HR reflects autonomic responses to environmental demands. In the presence of psychosocial stressors, HR typically increases due to vagal withdrawal and sympathetic activation [8–10]. Elevated HR has been linked to higher mental and social stress as well as increased anxiety [10, 11]. HRV reflects beat-to-beat fluctuations in heart rate and is a widely used marker of autonomic nervous system regulation influenced by both sympathetic and parasympathetic activity [12, 13]. Higher HRV indicates greater autonomic flexibility and more adaptive regulation of physiological arousal, particularly in social contexts [14, 15]. In contrast, psychosocial stress and anxiety are reliably associated with reduced HRV [16–18]. Beyond the lab, everyday social interactions are likewise crucial for regulating anxiety: In healthy individuals, engaging in social contact – particularly with familiar or trusted partners – is associated with lower levels of both state anxiety and state social interaction anxiety [19, 20].

Investigating the dynamic and context-dependent nature of social interactions in daily life requires a modern methodological approach. Ecological Momentary Assessment (EMA), also known as Ambulatory Assessment or Experience Sampling, is a research method that involves the repeated sampling of individuals’ behaviors, emotions, and experiences in real time and natural settings. Unlike traditional lab-based or retrospective self-report methods, EMA captures data with high resolution, temporal precision and ecological validity, allowing for a more nuanced understanding of how psychological and physiological processes unfold in daily life [21–23]. It is particularly beneficial in monitoring fluctuations in symptoms of depression and anxiety as they unfold over time, detecting subtle changes and offering insights that are both contextually and temporally grounded [24, 25]. Therefore, EMA helps overcome limitations of recall bias and cognitive distortions, particularly important in clinical populations prone to negatively skewed recall. Furthermore, the ambulatory nature of EMA might be especially important for examining the phenomenon of social buffering outside the artificial constraints of the laboratory, where the emotional relevance and ecological validity of interactions are limited. Overall, EMA enables the detailed analysis of how real-world social encounters, modulated by gender and familiarity, shape both subjective and physiological responses in a sample known to exhibit emotional dysregulation and autonomic inflexibility.

In light of these advantages, there is a growing number of studies using EMA to investigate the effect of social interactions on depression [26–30]. These studies demonstrate a dynamic, bidirectional relationship between mood and social behavior. Chronic depression is marked by persistent feelings of sadness, loss of interest in previously enjoyed activities, changes in appetite and sleep patterns, and diminished ability to think or concentrate [31, 32]. Common symptoms include restlessness, fatigue, irritability, muscle tension, and sleep disturbances [33]. A common comorbidity of depression is excessive fear or anxiety that is difficult to control and interferes with daily activities [34]. Besides enhanced levels of trait anxiety in general [35, 36] and with regard to social situations [37, 38], patients diagnosed with depression frequently show significantly higher levels of *state anxiety* [39] and *state social interaction anxiety* (State SI anxiety) [40], i.e., an increase in transient reactions to an adverse and social situation [41].

Although depression is commonly characterized by low positive affect and sadness, symptoms of anxiety are highly prevalent and clinically relevant in depression [42, 43]. Importantly, EMA research indicates that depression is not only associated with altered mean affect levels, but also with pronounced affective fluctuations and instability in daily life. Patients with current depressive or anxiety disorders often show greater variability in both positive and negative affect compared to remitted patients and healthy controls, even when controlling for mean affect levels [44, 45]. These findings highlight that affective processes in depression are dynamic and context-sensitive, underscoring the importance of examining momentary states such as anxiety, which may be particularly responsive to social and interpersonal contexts in daily life. Comorbidity rates between depression and anxiety disorders are high, and anxiety symptoms predict greater illness severity, functional impairment, and poorer treatment outcomes [34, 35]. Moreover, anxiety is particularly relevant to understanding social functioning in depression: elevated anticipatory and situational anxiety can interfere with engagement in supportive relationships and blunt the benefits of social buffering [38, 46]. While low positive affect and anhedonia are important features of depression, they tend to be more strongly reflected in altered baseline levels than in consistent, context-specific changes across social situations. In contrast, anxiety is dynamic and responsive to momentary interpersonal environments, making it a suitable primary outcome for EMA studies. In addition to cognitive and emotional symptoms, depression and anxiety are associated with dysregulation of the autonomic nervous system. Major depressive disorder is frequently linked to reduced autonomic flexibility, with lower baseline HRV and altered cardiovascular reactivity to stressors [47, 48]. Similarly, anxiety is often related to disorders that exhibit heightened sympathetic activation and reduced parasympathetic control, as reflected in increased resting HR and decreased HRV [49, 50]. Thus, focusing on anxiety allows us to capture in-the-moment affective processes and their autonomic correlates during real-world social interactions in patients with depression.

Recent EMA studies have focused on the complex interplay between social interactions and affective states. Geyer et al. [26] demonstrated that the relationship between negative affect during and the perception of social interactions was moderated by social anxiety and depression severity. With higher social anxiety negative affect had a stronger effect on the enjoyment of a social interaction, while higher depression moderated the link between negative affect and meeting the demands of a social interaction. Vranceanu et al. [30] reported that depressive symptoms were directly associated with increased negative affect and lower positive affect as well as indirectly through experiencing more social conflict. However, affect ratings may have reflected overall stress during assessment period, not solely social events. Additionally, these results may not generalize to individuals with clinical depression. Liu et al. [29] found that both quantitative (e.g., frequency) and qualitative (e.g., interpersonal perceptions) aspects of social interactions are moderately associated with fluctuations in positive and negative affect within individuals. Kamarsu et al. [27] extended these findings to bipolar disorder, showing that positive affect is higher and negative affect lower during social interactions, with social activity more strongly predicting subsequent mood states than vice versa. These results underscore the importance of social engagement in mood regulation and suggest that real-time monitoring of social behavior may provide valuable targets for therapeutic interventions aimed at improving social functioning and emotional well-being.

However, little is known about whether and how social interaction reduces different facets of anxiety in depressive patients. Given that negative cognitive biases often disrupt the encoding and storage of beneficial experiences in these populations, patients with depression and anxiety may show a disconnect between how they feel *in the moment* and how they later recall or evaluate their social interactions, undermining the internalization of positive social feedback [51]. Such distortions can perpetuate maladaptive patterns of avoidance and withdrawal, ultimately furthering the very conditions that impede recovery [52]. Given these biases and underlying physiological factors, it is possible that depression limits the capacity for social contact to reduce state anxiety and state SI anxiety in daily life.

Moreover, there is evidence that the effects of social interaction on anxiety depend on various factors, including the partner’s *familiarity* [19, 53], as well as characteristics of both the “actor” and the “partner” [20, 54]. Interacting with close friends or family members tends to bolster feelings of safety and reduce anxiety, whereas spending time with strangers or authority figures can heighten social anxiety and negative affect [55, 56]. Laboratory studies indicate that social familiarity and individual differences in autonomic regulation modulate cardiac responses and social information processing during face perception. Viewing faces of loved or personally familiar individuals elicits distinct cardiac responses compared to unfamiliar faces, characterized by increased heart rate and coordinated activation of brain regions involved in emotional and social processing, reflecting heightened autonomic engagement during the perception of socially salient stimuli [57]. Complementing these findings, research on resting vagally mediated heart rate variability (vmHRV) suggests that higher vmHRV is associated with a reduced tendency to perceive social evaluative threat from unfamiliar faces, as reflected in higher ratings of trustworthiness and caring [58]. Moreover, higher resting vmHRV has been linked to enhanced memory for faces associated with socially and affectively meaningful information, indicating more efficient integration of autonomic regulation and social cognition [59]. Together, these laboratory findings suggest that both social familiarity and autonomic regulation shape cardiac responses and social processing of faces, providing a theoretical basis for expecting familiarity-related differences in HR and HRV during real-world social interactions.

In addition, research on gender suggests that women and men may experience social buffering differently: some studies indicate that women exhibit stronger reductions in anxiety when a supportive partner is present, while other findings offer no clear gender differences [5, 6, 60]. Beyond main effects of participant gender, accumulating evidence suggests that the gender of the interaction partner, and the gender constellation of the interaction more broadly, plays an important role in shaping affective and physiological responses. Laboratory studies indicate that gender-dependent effects of social interaction are not uniformly stress-buffering but depend on the social role, evaluative context, and partner characteristics. For example, Gramer and Reitbauer [61] demonstrated that social support modulated cardiovascular reactivity differently in men and women during active performance tasks, with support attenuating reactivity in men but enhancing task-related cardiovascular activation in women, particularly in same-gender support contexts. Complementing these findings, experimental work on social evaluation shows gender-specific differences in regulatory effort and autonomic responses depending on whether feedback is delivered by a same- or opposite-gender partner [62]. Extending this work, more recent laboratory research indicates that affiliative interactions such as interpersonal touch can also elicit gender- and partner-specific physiological responses. Debrot et al. [63], for instance, found that touch from opposite-gender strangers increased physiological stress responses, indicated by reduced heart rate variability, particularly in women, underscoring the role of social ambiguity and evaluative threat rather than social buffering per se.

Consistent with these laboratory-based findings, recent EMA research demonstrates that participant gender and interaction partner gender jointly modulate anxiety-related autonomic responses in daily life, with mixed-gender interactions differing systematically from same-gender encounters [20]. However, these effects have not always been consistent across studies [64], highlighting the complexity of the interplay among participant gender, partner gender, and familiarity. Given that most EMA studies investigating factors that shape the effects of real-life social interactions on anxiety were conducted with healthy participants, it remains unclear whether similar effects are also found in patients with depression disorders.

In the present study, we applied EMA to assess the effect of every-day life interactions on state anxiety and state SI anxiety in patients diagnosed with depression. In more detail, inspired by previous research in healthy participants [20], we investigated the roles of participant gender, partner gender, and partner familiarity in shaping the different aspects of anxiety and related physiological responses (HR and HRV).

Based on previous work [20], we derived four hypotheses for the clinical sample. First, previous research indicates that individuals with depression often exhibit heightened social anxiety in social settings [46]. In addition to well-established mood-state distortion and negative memory biases, evidence suggests that these cognitive-affective biases influence real-time social perception [65, 66]. Thus, even though EMA reduces retrospective recall bias by capturing experiences in near real time, individuals with depression may still interpret and experience social interactions more negatively in the moment. On this basis, we hypothesized that the reduction of state anxiety and state SI anxiety in the presence of a familiar person might be less pronounced in patients compared to healthy participants (H1). Second, we hypothesized that the stronger decrease in HR and increase in HRV with increasing familiarity might be more pronounced in women compared to men, similar to the healthy sample [20, 60]. If this holds true, we should observe a significant two-way interaction between familiarity and gender, but no significant three-way interaction with group (H2). Third, we expected previously observed gender-specific effects in HR, for instance, higher HR in women when interacting with female partners [67], to remain consistent regardless of clinical status (H3). Finally, we tested whether the link between familiarity and HRV is different in patients compared to controls, given that individuals with depression often show altered baseline HRV (H4) [47–49].

## Methods

### Sample

We estimated required sample sizes via simulation with the R package *mixedpower* [68], using the openly available dataset from Gründahl et al. [20] as the data-generating model. For each candidate sample size (ranging from 30 to 120 participants in increments of 5), we ran 1000 iterations and defined power as the proportion of simulations yielding a significant effect. For state anxiety, we targeted the main effect of interaction partner familiarity (*b* = −0.16, *p* < 0.001), which indicated that a total sample size of *n* = 30 achieves 1−β > 0.90. For heart rate, we examined two interactions: participant gender × partner familiarity (*b* = −0.78, *p* < 0.001) and participant gender × partner gender (*b* = −4.72, *p* < 0.001); required sample sizes for these effects were *n* = 95 and *n* = 70, respectively, to reach the same power level. We conducted equivalent simulations for HRV, investigating the same two interactions, and found that total sample sizes of *n* = 50 (participant gender × partner familiarity; *b* = 0.05, *p* < 0.001) and *n* = 115 (participant gender × partner gender; *b* = 0.15, *p* = 0.002) are needed for 1−β > 0.90. In addition to these a priori simulations, simulation-based sensitivity analyses evaluating the detectability of the tested main and interaction effects under the final model specifications are reported in the Supplement.

The healthy control sample partially overlaps with the dataset reported in Gründahl et al. [20]. Specifically, the control participants stem from the same EMA study but are analyzed here to address distinct research questions focusing on group differences between healthy controls and patients with depression, including autonomic correlates of social interaction anxiety. The present manuscript includes a newly recruited clinical sample and examines hypotheses that were not addressed in the previous publication.

Between May 2023 and April 2024, we recruited 64 patients (56 with EMA and ECG-sensors and 8 with EMA only) from the Department of Psychiatry, Psychotherapy and Psychosomatic of the University Hospital Würzburg. During the study, patients received inpatient treatment and resided on the psychiatric ward on weekdays and at home at weekends. Weekend vs weekday was included as a control variable in the analyses. Inclusion criteria were a diagnosis of current affective disorder, and fluency in German. Exclusion criteria were psychosis, acute suicidality, cardiovascular diseases, current pregnancy, current suffering from a physical illness (e.g., chronic pain), intellectual disability, epilepsy, consumption of psychotropics substances during the last week, and factors that could affect the usage of a smartphone, i.e., reading or hearing difficulties that have not been corrected by aids such as glasses or hearing aids or problems with motor skills. All patients had received a clinical diagnosis prior to recruitment during inpatient treatment at the Department of Psychiatry of the University Hospital Würzburg. Diagnoses were made in routine care by board-certified psychiatrists based on comprehensive clinical assessment according to ICD-10 criteria. This assessment typically included a clinical diagnostic interview assessing the diagnostic criteria for an ICD-10 diagnosis (F32, F33, F31), psychiatric history, collateral history, behavioral observation, and review of medical records. Somatic causes of the depressive syndrome were excluded by physical and neurological examination, routine laboratory tests (including thyroid function, renal and liver function, blood cell count etc.) and MRI, if there was not a recent imaging done within the last 5 years prior the current admission. Diagnostic decisions were discussed and confirmed within interdisciplinary clinical teams including at least one certified psychiatrist as well as at least two psychiatry trainees, one senior psychologist, nursing staff and social workers as well as complementary therapists) and documented in the electronic patient records. Patients were treated as inpatients; thus, all of the patients suffered from a severe depressive episode at admission (F33.2, F32.2 or F31.4). For the purposes of the present EMA study, these existing clinical diagnoses were adopted without additional diagnostic interviews, re-evaluation, or formal assessment of inter-rater reliability. Of the recruited patients, 3 had technical problems, and 4 patients never reported interactions within the last 30 min and therefore did never activate the social interaction questionnaire (see “EMA survey”). Consequently, 57 usable patient datasets (28 females) remained for pre-processing, all diagnosed with depression (*n* = 9 in addition diagnosed with anxiety; *n* = 4 bipolar). The individuals with additional diagnoses were included because our focus was on depressive psychopathology within affective disorders, and our inclusion criteria targeted affective disorders encountered in routine clinical care. Ninety-five percent of patients reported regular use of antidepressants, 16% of antiepileptics, 18% of benzodiazepines, 7% of lithium, 54% of antipsychotics, 9% of non-opioid painkillers, and 2% of opioid painkillers. The control sample consisted of 57 gender-matched individuals from data collected by Gründahl et al. [20], resulting in a total sample size of *n* = 114 participants. Response rates in both patients and controls were comparable to those typically reported in EMA studies (meta-analysis: *M* = 79.19%, *SD* = 13.64%; Wrzus & Neubauer, 2023). Specifically, the mean response rate was 73.4% (*SD* = 24.7%) for patients and 89.7% (*SD* = 16.8%) for controls, *t*(81.5) = 4.82, *p* < 0.001. After data curation (see “Data analysis”), we analyzed 810 social interactions (i.e., an average of 14.2 per participant) in the clinical sample and 895 social interactions (i.e., an average of 15.7 per participant) in the control sample. The study protocol was approved by the ethics committee of the medical faculty of the University of Würzburg (vote #240/21) and complies with the Declaration of Helsinki. Only participants who gave informed written consent were included in the study.

### Procedure

First, eligible patients were visited by medical personnel, personally screened for participation criteria, and invited to a pre-session, during which they completed sociodemographic information and questionnaires. Next, a chest belt with an ECG sensor was attached (for details, see “Autonomic measures”), and the participant’s schedule for the five EMA measurement days was reviewed to adjust the 12-hour measurement time windows, starting one hour after their usual wake-up time. During the subsequent 5-minute baseline measurement, patients sat upright, facing a 16:9 computer screen at approximately 140 cm distance. They were instructed to relax while watching an audio-visual clip of an aquarium. Afterwards, participants practiced the correct use of the ECG belt, and the EMA questionnaire application (app) provided on a study smartphone. Under the supervision of the experimenter, they completed the EMA survey in two imaginary scenarios (social and non-social) before receiving the study materials. During the EMA period, patients carried the smartphone with them while wearing the ambulatory ECG sensor within the designated 12-hour time windows.

### Measures

#### Questionnaires

Prior to the EMA period, patients were asked about symptoms of depression using the 15-item short form of the Center for Epidemiologic Studies Depression Scale German: “Allgemeine Depressionsskala—Kurzform”, ADS-K [69], and their social interaction anxiety using the social interaction anxiety scale (SIAS) [70]. Several other questionnaires were also assessed and are reported for transparency; however, they were not part of the hypotheses and were collected only for exploratory purposes: The Multidimensional Scale of Perceived Social Support [71], Social Phobia Scale [72], State-Trait Anxiety Inventory (STAI) [73], and the NEO Five Factor Inventory for the assessment of personality dimensions [74, 75].

#### EMA survey

The EMA surveys were administered via the movisensXS app (movisens GmbH) on an android study smartphone. In Gründahl et al. [20], state anxiety was assessed with a 10-item short form of the STAI state anxiety subscale on a Likert scale from 1 (“not at all”) to 8 (“totally”). In the clinical sample, we aimed at reducing patients’ burden and used only one item for state anxiety (“I feel anxious”) on a Likert scale from 1 (“not at all”) to 9 (“totally”). The differences in the answering format were adjusted through a linear transformation for interpretable descriptive statistics (see Results).

The EMA protocol followed a signal-based sampling design, with participants prompted up to six times per day over five consecutive days, to balance ecological validity and participant burden and to ensure comparability with prior work (Gründahl et al. [20]). At each prompt, participants first indicated whether a social interaction had occurred within the previous 30 min (“now”, “within the last 30 min”, “more than 30 min ago”). Reporting social interactions ≤ 30 min ago activated the social interaction questionnaire. Alternatively, they were directed to a similarly structured activity questionnaire to prevent strategic answers to shorten the survey duration. The use of alternative questionnaires is consistent with methodological recommendations in EMA research to minimize participant burden and reduce the likelihood of nonresponse or disengagement [76, 77].

Social interactions were broadly defined and could include direct face-to-face contact, telephone calls, or mediated forms of communication (e.g., text-based or online interactions). The social interaction questionnaire assessed the type (e.g., “direct contact”, “private”, “job-related”, etc.), the approximate start and duration of the social interaction (“ < 1 min”, “15 min”, “ > 30 min”), the quantity (“1” to “5 or more”) and gender (“female”, “male”, “mixed”) of the interaction partner(s). Moreover, we asked for the familiarity of the interaction partners (“I know the other person/one of the other persons well.”) using a Likert scale ranging from 1 (“not at all”) to 8 (“very”) [78]. State social interaction anxiety (state SI anxiety) was calculated as the average from two items (“I worried about what the other person(s) thought of me”, “I was worried that I would say or do the wrong things”) rated on a Likert scale from 1 (“not at all”) to 9 (“very”) [79, 80]. We also assessed the pleasantness of the interaction (“How (un)pleasant was the interaction?”) on an 8-point Likert scale ranging from 1 (“very unpleasant”) to 8 (“very pleasant”) [81]. Finally, we assessed ECG control variables e.g., consumption of caffeine/nicotine/alcohol during the last hour [82].

#### Autonomic measures

HR and HRV were continuously recorded using the ambulatory movisens EcgMove4 sensor (movisens GmbH, Karlsruhe, Germany). HR (beats per minute, bpm) increases under stress due to vagal withdrawal and sympathetic activation e.g., [9]. HRV, a marker of autonomic nervous system activity, reflects both sympathetic and parasympathetic activity [13]. It measures fluctuations in time intervals between heartbeats, with higher HRV linked to better stress regulation, engagement, and adaptive HR control [50].

Here, we used the root mean square of successive differences between heartbeats (RMSSD) measured in ms, which estimates vagal cardiac control and short-term parasympathetic variations. RMSSD, a robust time-domain HRV measure, is less affected by motion artifacts than frequency-domain measures and is suitable for both long- and short-term analyses [83, 84].

The ECG sensor, attached via a chest belt at the sternum base with two dry electrodes, required skin cleaning with alcohol pads (70% isopropyl alcohol) before placement. It recorded single-channel ECG data (12-bit resolution, 1,024 Hz sampling rate). Participants avoided heavy meals, coffee, alcohol, tobacco, and limited their water intake two hours before the baseline session [82]. Baseline ECGs were collected between 12 and 6 pm.

### Data analysis

#### ECG data curation and heart rate variability calculation

We used the movisens DataAnalyzer software (version 1.13.8) to convert the ECG signals into HRV indices. Prior to automated processing, ECG recordings were visually inspected to identify segments with excessive noise or implausible signal patterns. The DataAnalyzer applies automated algorithms for R-peak detection and artefact identification based on the raw ECG signal. R-peak detection was adapted from Hamilton [85]. Artefact detection was based on physiological plausibility criteria, including signal amplitude and the number of zero crossings per second outside a normal range. Artefact filtering followed established recommendations [86] and checked for valid changes in consecutive RR intervals and R-peak amplitudes. Interbeat intervals outside the range of 250–2000 ms and R-peak amplitudes outside the range of 0.1–5.0 mV were removed, producing an NN-interval list. The maximum allowed variation was set to 20% for RR intervals and 30% for R-peak amplitudes. RR-interval lists consisted of overlapping 2-minute segments with 30-second shifts; segments with an insufficient number of valid NN intervals were excluded. To meet stationarity criteria for subsequent HRV calculation, NN intervals were detrended [87]. HR and HRV indices were computed using minute-by-minute calculations derived from the 2-minute segments. Raw acceleration data were averaged over 30-second periods, from which mean acceleration per 60-second epoch was derived. ECG and EMA data streams were time-aligned using the movisens DataMerger software.

Afterwards, we rounded ECG data to whole-minute values, excluded ECG segments labelled as invalid, and calculated mean scores for HR and RMSSD at baseline (first minute excluded). We calculated separate outlier detections for baseline and EMA values of HR and RMSSD. Values outside the upper and lower quartile by at least 1.5 times the interquartile range were removed. Subsequently, we extracted mean HR and RMSSD values for each social interaction (time retrieved from slider scales, transformed to 1-min-intervals; first and last segment of time window excluded). The survey prompt marked the end of ongoing social interactions. We removed social interactions shorter than 60 s. A natural logarithmic transformation was performed on RMSSD values to correct for skewness.

Due to excessive artifacts and missing data, only 24 datasets remained for analysis. Specifically, post-hoc visual inspection revealed excessive artifacts in the baseline ECG measurements of 25 patients, leading to their exclusion. Among the patients with valid baseline measurements, data collection during the EMA period failed to yield valid data for an additional 18 patients, largely due to compliance issues, such as belts not being worn properly or sensors not being recharged as required. Thus, for the analyses including ECG data, we combined these 24 valid data sets from the patients with 24 gender-matched participants from the control sample to keep sample sizes balanced. However, there were no significant differences between included and excluded participants on key measures, such as the ADS (included: M = 21.58, SD = 8.34; excluded: M = 23.58, SD = 10.98; t(54.9) = −0.78, *p* = 0.440) or the SIAS (included: M = 46.79, SD = 14.11; excluded: M = 46.45, SD = 19.45; t(55.0) = 0.08, *p* = 0.940). Gender distribution was also comparable across groups (χ²(1) ≈ 0.00, *p* = 1.000), with roughly equal proportions of male (50.0% vs. 51.5%) and female (50.0% vs. 48.5%) participants in the included and excluded subsamples, respectively. Taken together, these findings indicate that excluding participants with invalid ECG data did not introduce systematic bias in key symptom measures or gender composition.

#### Statistical analyses

To test our hypotheses, we calculated four linear mixed models with state anxiety, state SI anxiety, HR, and RMSSD as dependent variables, respectively, and participant as random intercept. Model significance was calculated with the ‘lmerTest’ package [88], applying Satterthwaite’s method to estimate degrees of freedom and *p*-values. In all models, continuous within-person variables were person-centered and continuous between-person variables were group or grand mean-centered [77]. All models were fully specified based on a priori hypotheses, with predictors and interaction terms entered simultaneously.

For the state anxiety and state social anxiety models, we specified full mixed-effects models including the following predictors to address our hypotheses: group (healthy controls vs. patients), participant gender (female, male), familiarity of the interaction partner (centered within [cw]), gender of the interaction partner (female, male, mixed), and social interaction type (direct, virtual). Control variables [82, 84] were age (centered between [cb]), type of day (weekday vs. weekend), duration of the social interaction (in seconds, cw), number of interaction partners (1 vs. 2 or more), and recent caffeine, nicotine, and alcohol consumption (each coded yes/no). We also included trait social interaction anxiety (SIAS; centered within group [cg]) and depressive symptoms (ADS-K; cg) as between-person covariates to reduce potential confounding by stable individual differences. Both constructs may influence the type of interaction partners encountered (e.g., selecting more familiar partners or avoiding challenging interactions). Controlling for SIAS and ADS-K therefore allowed us to more specifically estimate within-person associations between social-context variables (e.g., partner familiarity) and momentary outcomes. We added interactions between group and ADS-K, group and SIAS, and group and type of day, as well as higher-order interactions between group, participant gender, and gender of the interaction partner, and between group, participant gender, and familiarity. Additional exploratory analyses examining the interaction between interaction pleasantness, partner familiarity, and group are reported in the Supplement. All categorical predictors were effect coded to facilitate interpretation of main effects and interactions and to reduce multicollinearity (as indicated by VIF). For the factor *gender of interaction partner* (female, male, mixed), we created two orthogonal contrasts: one comparing interactions with mixed-gender partners to those with exclusively female or male partners, and another comparing male versus female partners while ignoring mixed-gender interactions. Other categorical predictors with two levels (e.g., group, participant gender, type of day) were coded using ±0.5 coding. Likelihood ratio tests supported including a random slope for type of day, so we report random-slope models.

For the HR and HRV models, we used the same overall modeling strategy but simplified the specification due to the reduced dataset (*n* = 48 participants) to prevent overparameterization. Specifically, we removed between-person variables (SIAS, ADS-K, and age) and secondary social interaction variables (number of interaction partners, interaction duration, and interaction type). The final ECG models therefore focused on group, participant gender, gender of the interaction partner(s), type of day, and familiarity of the interaction partner(s). We specified all relevant two-way interactions (familiarity × participant gender, familiarity × gender of interaction partner(s), familiarity × group, participant gender × group, group × type of day) and three-way interactions (group × participant gender × gender of interaction partner(s), group × participant gender × familiarity). To account for ECG-specific considerations, these models also included mean movement acceleration (centered within [cw]) and baseline HR or HRV (centered between [cb]) as recommended for ECG analyses [82].

We performed complete-case analysis by omitting observations with missing values on any variables included in the models. We applied a Bonferroni correction across the four outcome variables (state anxiety, state social anxiety, HR, and HRV) to account for multiple testing, such that all parameter p-values were multiplied by four. We report significant results after Bonferroni correction. Complete results, including all fixed-effect estimates with both model-based and robust standard errors, are presented in the Supplement.

## Results

### Descriptive statistics

Depression, social interaction anxiety, HR at baseline and during EMA were higher in the clinical sample (all values of *p* < 0.001), whereas familiarity of the interaction partner, pleasantness of the interaction, HRV at baseline and during EMA were higher in the healthy sample (all values of *p* ≤ 0.002). On average, the patient sample was older compared to the healthy control sample (33 vs. 26 years, *p* = 0.001). The self-reported state anxiety (*p* = 0.374) and state SI anxiety (*p* = 0.727) did not differ between samples (for details, see Table 1). Patients predominantly reported interactions with female partners (57%), followed by mixed (22%) and male (21%) partners. Participants in the control group reported 38% interactions with female partners, 32% with male partners, and 30% with mixed partners. Across both groups, the majority of interactions took place in person. Specifically, 90% of interactions in the patient group and 82% in the control group were personal face-to-face interactions, whereas 10% (patients) and 18% (controls) were categorized as other interactions (i.e., telephone call, e-mail, letter, SMS, and social media). Patients reported most interactions with their partners (57%), followed by acquaintances (13%), friends (10%), family (6%), strangers (6%), medical staff (5%), therapists (3%) and colleagues (2%), and participants in the control group reported most interactions with their friends (32%), followed by partners (27%), family (16%) and colleagues (16%), strangers (6%) and acquaintances (4%). In addition, 65% of the patient interactions occurred on weekdays, whereas 58% of the control group interactions took place on weekdays.

**Table 1:** Characteristics of the healthy control and the patient sample.

	Control [*n* = 57, 29 females]		Patients [*n* = 57, 28 females]		Comparison	
	Mean	SD	Mean	SD	*t*-value	*p*-value
Age	25.67	4.54	33.14	15.34	−3.53	0.001
ADS-K	10.04	3.99	22.74	9.92	−8.97	< 0.001
SIAS	20.98	11.35	46.60	17.26	−9.36	< 0.001
State SI Anxiety	2.59	1.25	2.51	1.33	0.35	0.727
State Anxiety	2.84	1.23	2.62	1.40	0.89	0.374
IP familiarity	6.09	1.19	3.37	1.21	12.11	< 0.001
SI pleasantness	6.01	0.83	5.24	1.09	4.56	< 0.001
	Control (12 females)		Patients (12 females)			
HR [baseline]	68.89	13.35	92.08	12.06	−6.32	< 0.001
RMSSD [baseline]	54.70	26.16	24.20	22.88	4.30	< 0.001
HR [EMA]	81.28	7.54	93.66	11.02	−4.54	< 0.001
RMSSD [EMA]	37.59	7.05	22.37	20.59	3.42	0.002

For the EMA data, the sample size was *N* = 57 for both the control participants and patient sample, whereas for the ECG data, the sample size was *N* = 24 for each group.

*ADS-K* Allgemeine Depressionsskala (depression scale), *SIAS* social interaction anxiety scale, *SI* social interaction, *HR* heart rate, *RMSSD* root mean square of successive differences between heartbeats.

### State anxiety

**Fig. 1:**
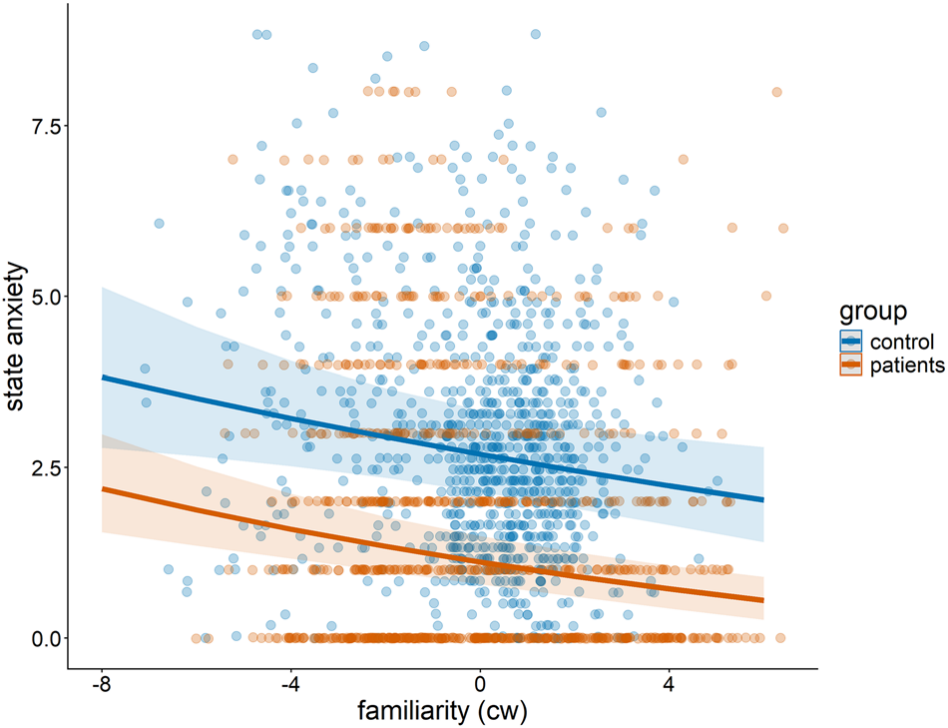
Non-significant interaction effect of familiarity of the interaction partner and participant group (controls vs. patients) on state anxiety ratings. The main effect of familiarity was statistically significant. Each datapoint represents raw data from a single observation, and the shaded areas indicate the standard errors of the predicted values.

In the log-transformed state anxiety model (marginal R^2^ = 0.337), there was a main effect of group (B = 0.532, *p*_*adj*._ < 0.001), surprisingly indicating that controls had *higher* state anxiety. Additionally, there was a main effect of familiarity (*B* = − 0.039, *p*_*adj*._ < 0.001), reflecting a decrease in state anxiety with increasing familiarity of the interaction partner. However, there was no interaction between familiarity and the group (*B* = 0.01, *p*_*adj*._ = 1.000, see Fig. 1), indicating that the strength of this effect did not differ between groups. Further main effects showed higher scores on the SIAS and ADS-K were associated with higher state anxiety (SIAS: *B* = 0.012, *p*_*adj*._ < 0.001; ADS-K: *B* = 0.025, *p*_*adj*._ < 0.001), and alcohol consumption was associated with lower state anxiety (*B* = −0.140, *p*_*adj*._ = 0.016)

**Fig. 2:**
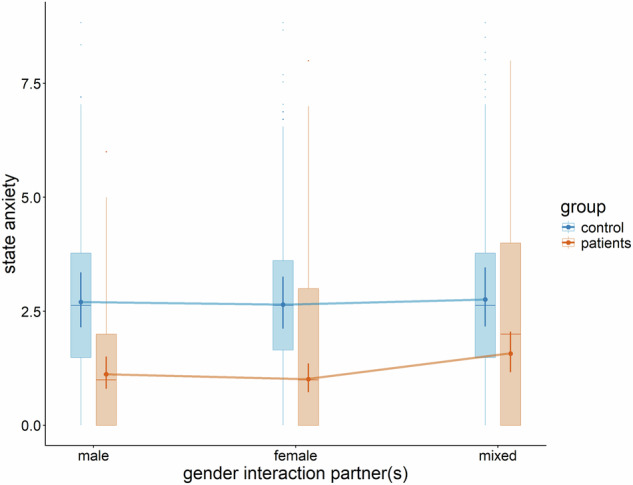
Significant interaction effect of gender of interaction partner(s) and participant group (controls vs. patients) on state anxiety ratings. Dots and error bars are the estimated marginal means of the linear mixed effects model, boxplots represent the raw data.

Moreover, there was an interaction between group and the contrast of mixed interaction partners compared to exclusively male or female interaction partner(s) (*B* = −0.276, *p*_*adj*._ = 0.004, see Fig. 2). A Holm-adjusted post-hoc test between all levels of gender of interaction partners for each group revealed significantly less state anxiety with male interaction partners compared to mixed interaction partners for patients (difference = −0.275, *t*(1607) = −3.36, *p*_*adj*._ = 0.005) and lower state anxiety in interaction with female interaction partners compared to mixed interaction partners for patients (difference = −0.213, *t*(1605) = −2.79, *p*_*adj*._ = 0.027). All other comparisons were not significant. All other fixed effects of the model were not significant (all values of *p*_*adj*._ ≥ 0.096). All fixed effects are reported in the Supplementary Material.

The residual variance was σ² = 0.17. The random intercept variance at the participant level was τ₀₀ = 0.13, and the random slope variance for type of day was τ₁₁ = 0.05, with a correlation between intercept and slope of ρ₀₁ = –0.14. The intraclass correlation coefficient (ICC) was 0.45.

### State social interaction anxiety

For log-transformed state SI anxiety (marginal R^2^ = 0.227) type of day had a significant main effect (*B* = 0.117, *p*_*adj*._ = 0.004), indicating higher state SI anxiety on weekdays compared to the weekend. There was also a significant relation between higher SIAS-scores and higher state SI anxiety (*B* = 0.014, *p*_*adj*._ < 0.001). Social interactions with multiple partners were associated with higher social anxiety as interactions with individuals (*B* = 0.365, *p*_*adj*._ < 0.001). The contrast between interactions with female interaction partners vs. male interaction partners indicated higher social state anxiety in interactions with males (*B* = 0.162, *p*_*adj*._ = 0.004).

**Fig. 3:**
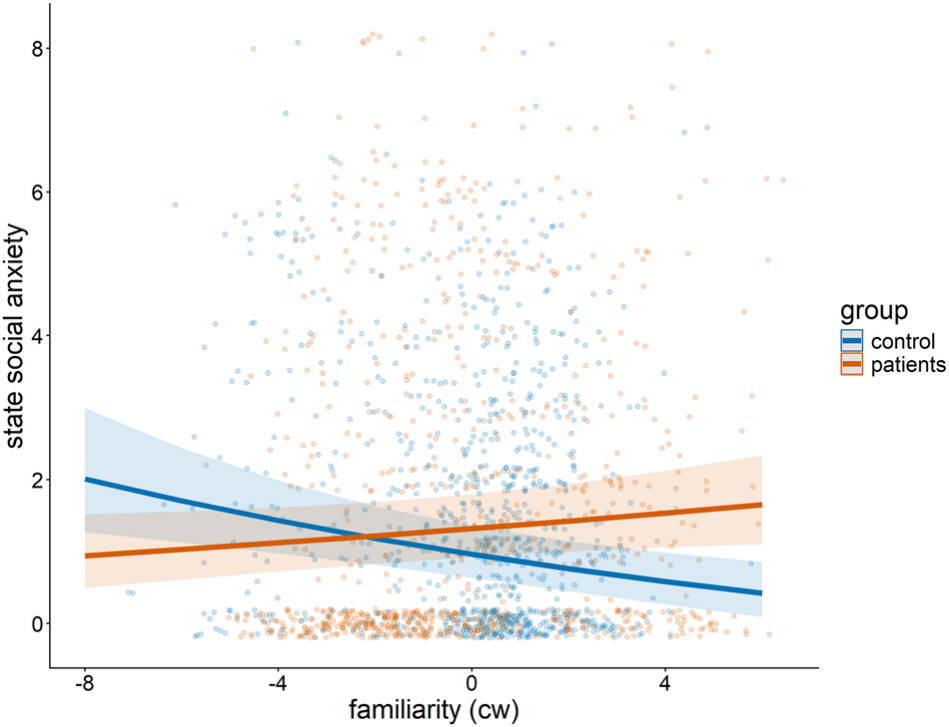
Significant two-way interaction between familiarity of the interaction partner(s) and group (controls vs. patients). Each datapoint represents raw data from a single observation. Lines and error bars are predicted from the linear mixed effects model.

**Fig. 4:**
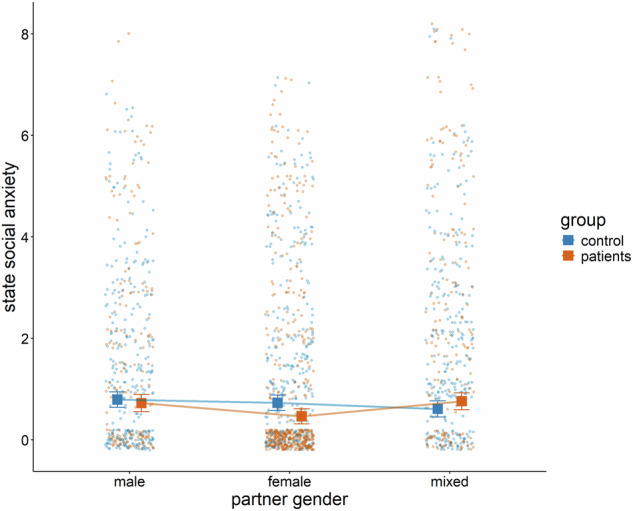
Significant two-way interaction between gender of the interaction partner(s) (female, male, mixed) and group (controls vs. patients). Each datapoint represents raw data from a single observation, and the error bars indicate the predicted mean along with its robust confidence intervals.

Additionally, the interaction between familiarity and the group (patients vs. controls) was significant (*B* = −0.068, *p*_*adj*._ = 0.024). Holm-adjusted simple slopes analyses showed that controls experienced significantly less state SI anxiety when the interaction partner was more familiar (*B* = −0.048, *t*(1576) = −2.61, *p*_*adj*._ = 0.018) while there was no significant association between familiarity and state SI anxiety for patients (*B* = 0.021, t(1157) = 1.33, *p*_*adj*._ = 0.183, see Fig. 3). Moreover, there was a significant interaction between the contrast of mixed vs. non-mixed genders of the interaction partner(s) and group (*B* = −0.320, *p*_*adj*._ = 0.004, see Fig. 4). Holm-adjusted post-hoc pairwise comparisons between both groups for each level of gender of interaction partner(s) revealed higher state social anxiety when controls interacted with females compared to when patients interacted with females (difference = 0.267, *t*(139) = 2.89, *p*_*adj*._ = 0.013). All other comparisons between controls and patients were not significant. No other fixed effects were significant (all values of *p*_*adj*._ ≥ 0.080). All fixed effects are reported in the Supplementary Material.

The residual variance was σ² = 0.24. The participant-level intercept variance was τ₀₀ = 0.15, and the slope variance for type of day was τ₁₁ = 0.09, with an intercept–slope correlation of ρ₀₁ = –0.16. The ICC was 0.41.

### Heart rate

**Fig. 5:**
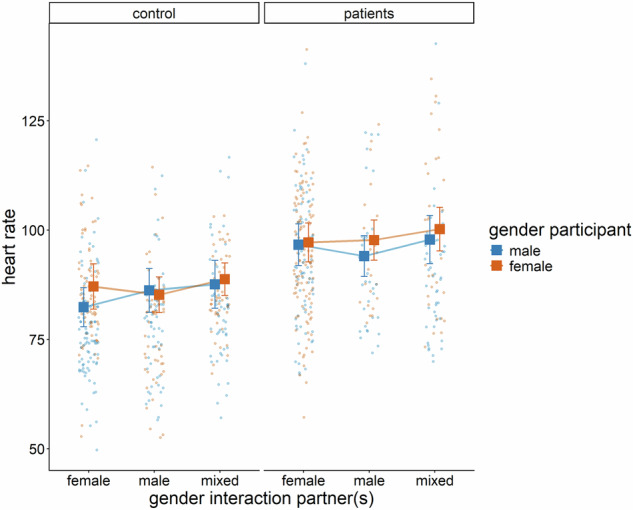
Significant three-way interaction between participant group (controls vs. patients), participant gender (male vs. female), and gender of the interaction partner(s) (female, male, mixed) on heart rate. Each datapoint represents raw data from a single observation, and the error bars indicate the predicted mean along with their robust confidence intervals.

In the HR model (marginal R^2^ = 0.587), group had a significant main effect (*B* = −11.08, *p*_*adj*._ < 0.001), indicating lower HR in controls compared to patients. Acceleration was positively associated with HR (*B* = 114.96, *p*_*adj*._ < 0.001). Baseline HR also showed a significant positive association with heart rate (*B* = 0.46, *p*_*adj*._ < 0.001). Finally, interactions with mixed-gender partners were associated with higher HR compared to interactions with only male or female partners (B = 2.80, *p*_*adj*._ = 0.004). Additionally, we found a significant three-way interaction between group, gender of the participant and the contrast between male minus female interaction partners (*B* = −8.89, *p*_*adj*._ < 0.024, see Fig. 5). Holm-adjusted post-hoc pairwise comparisons between levels of gender of interaction partner(s) within each combination of participant gender and group showed that, among male controls, HR was significantly higher during interactions with mixed-gender partners compared to female partners (difference = 5.23, *t*(629) = 4.06, *p*_*adj*._ = 0.001). Among female controls, HR was significantly higher during interactions with mixed-gender partners compared to male partners (difference = 3.56, *t*(647) = 2.98, *p*_*adj*._ = 0.033). All other comparisons were not significant.

No other fixed effects were significant (all values of *p*_*adj*._ ≥ 0.068). All fixed effects are reported in the Supplementary Material.

The residual variance was σ² = 49.93. The participant-level intercept variance was τ₀₀ = 36.91, and the slope variance for *type of day* was τ₁₁ = 20.31, with an intercept–slope correlation of ρ₀₁ = –0.03. The ICC was 0.46, based on 48 participants.

### Heart rate variability

For heart rate variability (marginal R^2^ = 0.592) group had a significant main effect (*B* = 0.782, *p*_*adj*._ < 0.001), indicating higher HRV in controls compared to patients. Acceleration was negatively associated with HRV (*B* = –2.67, *p*_*adj*._ < 0.001). Baseline HRV showed a significant positive association with HRV (*B* = 0.538, *p*_*adj*._ < 0.001).

None of the interactions and no other main effects were significant (all values of *p*_*adj*._ ≥ 0.060).

The residual variance was σ² = 0.11. The participant-level intercept variance was τ₀₀ = 0.13, and the slope variance for *type of day* was τ₁₁ = 0.05, with an intercept–slope correlation of ρ₀₁ = –0.32. The ICC was 0.56, based on 48 participants.

## Discussion

The present study investigated how familiarity, participant gender, and interaction partner gender influenced state anxiety, state social interaction anxiety, and autonomic responses (HR and HRV) in healthy controls and patients with depression using an EMA approach.

We hypothesized that the reduction of state anxiety and state SI anxiety in the presence of a familiar person might be less pronounced in patients compared to healthy participants. Confirming this hypothesis, state anxiety decreased with increasing familiarity of the interaction partner in both patients and controls (Fig. 1). Although group differences in absolute levels of state anxiety should be interpreted cautiously, as different instruments were used in patients and controls, the within-person associations between social context variables and anxiety showed comparable patterns across groups. In contrast, state SI anxiety decreased with greater interaction partner familiarity only in healthy control participants, whereas no such effect was observed in patients (Fig. 3). This finding is consistent with theoretical accounts suggesting that depression dampens the benefits of familiar social connections [26, 89]. Extending these previous findings, our results show that these dampening effects are specific for state SI anxiety. For general state anxiety, patients with depression appear to retain sensitivity to the emotional buffering provided by familiar others. This suggests a specific association between depressive symptoms and state SI anxiety, but not necessarily with state anxiety. One possible explanation for this distinction is that patients with depression often report feelings of guilt or shame in interactions, sometimes unrelated to the social context [90]. Healthy controls, on the other hand, may be better able to differentiate between familiar and unfamiliar contexts, which could reduce state SI anxiety when interacting with familiar individuals. Additionally, previous research has linked depressive symptoms to higher sensitivity for social rejection [91].

In addition to the observed effects of interaction partner familiarity, the gender of the interaction partners had differential effects on state anxiety and state social anxiety in patients and controls. In more detail, in patients, state anxiety was higher during interactions with mixed-gender partners (i.e., when interacting with both males and females) compared to interactions exclusively with males or females. In contrast, for controls, the gender composition of interaction partners had no evidence for an association with state anxiety. This finding aligns with prior research indicating that depressive symptoms are associated with a heightened likelihood of engaging in pairwise rather than larger-group social interactions, a phenomenon termed *dyadic isolation* [92]. Importantly, this tendency appears to be primarily driven by a preference among individuals with depressive symptoms for dyadic over group interactions, rather than by avoidance on the part of potential interaction partners [93]. Critically, such behavioral patterns may exacerbate depressive symptoms, as dyadic isolation has been shown to foster co-rumination [94, 95] and negative affective contagion [96]. In turn, heightened depressive symptoms may further reinforce dyadic isolation, potentially giving rise to a self-perpetuating vicious cycle [97].

Moreover, results revealed higher state social anxiety in controls compared with patients when interacting with females. This finding is potentially due to the different nature of interactions with female interaction partners. Overall, patients reported most interactions with their partners (57%), while controls mainly interacted with their friends. Social evaluation or comparison inducing social SI may be stronger when interacting with friends or peers, compared to regular partners [98, 99], resulting in a social anxiety inducing effect of females in controls, but not in patients.

In summary, these findings demonstrate the importance of differentiating between anxiety related constructs and their relation to social interactions. Our study is the first to test the effects of everyday-life familiarity on state anxiety and state SI anxiety in both patients with depression and healthy controls.

Regarding autonomic responses, we hypothesized that familiarity of the interaction partner might be associated with a stronger decrease in HR and increase in HRV, with potentially stronger effects in females compared to males [20, 60]. Moreover, we expected previously observed gender-specific effects in HR, for instance, higher HR in women when interacting with female partners [67], to remain consistent regardless of clinical status.

We found no statistical evidence for an association between familiarity and heart rate or heart rate variability. While we observed a significant three-way interaction involving group and gender on HR, the hypothesized two-way gender-related effects were not supported, which may reflect small true effects and/or limited power and precision due to the reduced sample size. Specifically, control participants, but not patients, showed lower HRs when interacting with opposite-gender partners (i.e., male controls with female partners and female controls with male partners) compared to interactions with mixed-gender groups. These findings are in line with previous research in healthy participants, showing that women experience reduced physiological stress (lower HR) when interacting with male partners, even in the absence of a romantic relationship [20]. In control participants, interactions with female and male interaction partners were relatively balanced (38%/ 32%), while patients predominately interacted with females (57%) and less frequently with males (21%). The lack of the gender effect in patients might be due to this imbalance between female and male interaction partners. Alternatively, they may reflect depression-specific alteration in physiological responding. In line with this assumption, patients exhibited higher HR and lower HRV than controls, both at baseline and during social interactions. This finding aligns with well-documented autonomic dysfunction in depression and anxiety in previous research [50] and might indicate sympathetic hyperactivity, reflecting a reduced ability to physiologically regulate stress [47, 100, 101].

Finally, our findings should be interpreted in light of ecological research highlighting the importance of contextual moderators for autonomic regulation in daily life. For example, Bailey et al. [102] demonstrated that the valence of social interactions moderates the autonomic consequences of perseverative cognition in individuals with major depression and social anxiety disorder, such that reduced HRV following perseverative thinking was particularly pronounced after negative social interactions. Although the present study did not focus on perseverative cognition, these findings underscore the broader relevance of social-contextual factors when examining autonomic responses in everyday life.

### Limitations and future directions

Patients reported consistently lower state anxiety than healthy controls. This might be related to the protected setting of a clinical ward, where patients may have felt more secure and somewhat shielded from external stressors such as (care-)work, or may have experienced a sense of safety due to structured daily routines. Additionally, these absolute differences in state anxiety between groups should be interpreted with some caution. Our healthy control sample was drawn from another study (Gründahl et al. [20]), where state anxiety was assessed using the 10-item STAI (Laux et al. [73]), whereas our patient group responded to a single-item question. Although both measures are closely related, they may operationalize the construct differently, potentially resulting in variations in mean scores. Importantly, however, we showed similar dynamics in both measures regarding their association with familiarity of the interaction partner. While internal consistency cannot be estimated for single-item measures, recent EMA studies indicate that single-item affect ratings demonstrate adequate concurrent and predictive validity in intensive longitudinal designs, with only modest advantages of multiple-item measures [103].

Several limitations related to the EMA design should also be acknowledged. The signal-based sampling approach may have resulted in the omission of particularly salient or emotionally intense social interactions, as assessments were prompted at predefined time points rather than contingent on interaction events, a trade-off commonly discussed in EMA research [104, 105]. Moreover, because participants reported either on a very recent social interactions or on ongoing activities, some social interactions may not have been captured. Social interactions were broadly operationalized and included direct face-to-face contact, telephone calls, and mediated forms of communication. While this inclusive definition enhances ecological validity, it also introduces heterogeneity in interaction modalities that may differ in their interpersonal and sensory characteristics. However, as reflected in the descriptive statistics, the majority of reported interactions involved personally relevant partners (e.g., partners, friends, family, or acquaintances), whereas interactions with more distal or institutional contacts (e.g., medical staff, therapists) occurred relatively infrequently. Thus, although different interaction modalities were allowed, less interactive or contextually specific contact types likely played a subordinate role in the overall pattern of results. In addition, interaction partner familiarity was assessed as a continuous, subjective rating, which likely captures heterogeneous relationship types (e.g., romantic partners, family members, friends, colleagues, or acquaintances). While this approach allows participants to integrate multiple dimensions of closeness and trust, it does not distinguish between qualitatively different relationship categories that may differentially shape anxiety and autonomic responses. Furthermore, social interactions involving single versus multiple partners likely differ in their interpersonal dynamics. In group interactions, information was collected on predominant group characteristics (e.g., familiarity or gender composition) rather than on a specific focal interaction partner. By doing so, we captured the effects of group characteristics and were able to include situations in which participants might have interacted with several individual simultaneously. Asking about the primary interaction partner would capture the effects of interactions with one specific group member, which is another interesting aspect that should be investigated in future studies.

According to sensitivity analyses (see Supplement), small-to-moderate familiarity-related main effects and Group × Familiarity interaction effects in the self-reported outcomes (state anxiety and state social interaction anxiety). Thus, both the observed familiarity effects and the absence of group moderation for state anxiety can be interpreted with reasonable confidence, as effects of theoretically meaningful magnitude would have been detectable under the present design. For physiological outcomes, power was sufficient to detect clinically meaningful main effects of group on heart rate and heart rate variability, which were robustly observed. Sensitivity for detecting main effects of familiarity was also adequate for heart rate and heart rate variability. In contrast, substantially larger effect sizes were required to achieve adequate sensitivity for higher-order interaction effects in the autonomic models, indicating limited precision for detecting subtle two-way and three-way interactions in these outcomes. Thus, the absence of significant two-way and three-way interactions on HR and HRV has to be interpreted with caution because of insufficient statistical power. The significant three-way interactions between group (patient vs. control), participant gender, and gender of the interaction partners on HR likely reflects a robust effect that was large enough to be detected despite our reduced sample size. More generally, the effective number of EMA observations per participant was lower than the maximum possible number of prompts, which reduced power and precision for detecting cross-level and higher-order interaction effects. Therefore, non-significant interaction terms should be interpreted cautiously and not as evidence of the absence of effects. Future studies with larger ambulatory ECG samples and/or longer sampling periods are needed to more reliably detect small-to-moderate interaction effects in daily-life autonomic responses.

Patients received inpatient treatment and resided on the psychiatric ward on weekdays. The inpatient setting is characterized by structured daily routines, increased professional support, and reduced exposure to external stressors (e.g., work or family demands), which may have influenced baseline levels and moment-to-moment variability of anxiety during social interactions. Although weekday versus weekend was included as a covariate, such contextual factors may still have shaped patients’ emotional experiences in daily life and could partly contribute to the observed group differences in state anxiety. However, they were at home at weekends and the majority of patients’ interactions involved their partners (57%), and acquaintances (13%), which were the two most frequent groups of interaction partners also in the control group. In comparison, the percentage of patients’ interactions with clinical personnel was relatively small (medical staff = 5%; therapists = 3%. Thus, although patients were in a clinical setting, overall the type of interaction partners was comparable to the control sample and also contained interactions in other contexts. Future research should explore long-term effects, individual differences, and recovery patterns to refine interventions aimed at enhancing social and autonomic resilience in anxiety and depression.

## Conclusion

In summary, this study highlights the role of familiarity in reducing state and social anxiety in both healthy individuals and patients with depression. Patients benefited from familiar interactions similarly to controls in terms of general state anxiety. However, only controls reported lower state social interaction anxiety when the familiarity of interaction partners was high. Among patients, the association between familiarity and state social interaction anxiety was not statistically reliable. This lack of statistical evidence does not necessarily indicate the absence of an effect, but may reflect differences in how interpersonal contexts are appraised in depression. At the same time, alternative explanations, including limited statistical power, measurement differences, or contextual differences in interaction partners, remain plausible.

Patients exhibited elevated heart rate and reduced heart rate variability, confirming autonomic dysregulation in clinical populations. Gender differences in heart rate depending on the participants’ gender and the gender of the interaction partners only occurred in control participants, but not in patients. Our findings add to a growing body of EMA research and emphasize the potential of momentary social encounters to support emotional and physiological regulation, even in clinical populations.

## Supplementary information

Supplementary Material

## Data Availability

The datasets generated and/or analyzed in the current study are not publicly available due to the sensitive nature of patient data. However, they can be obtained from the corresponding author upon reasonable request.

## Code availability

The analysis code used in this study is publicly available on the Open Science Framework (OSF) at https://osf.io/jctyg.
