# Supplementary material for: Momentary anxiety and autonomic responses during everyday social interactions among patients with depression
Source: Transl Psychiatry. 2026 Apr 4;16:234. doi: 10.1038/s41398-026-03990-y (PMC13079786; doi:10.1038/s41398-026-03990-y)
Supplement: Supplementary file 1 — Supplementary Material [file 41398_2026_3990_MOESM1_ESM.docx]

# Supplementary Material

**Momentary anxiety and autonomic responses during everyday social interactions among patients with depression**

Weiß, M.^1, 2, *^, Gutzeit, J.^2^, Jachnik, A.^2^, Lampe, E.C.^2^, Rothbauer, F. ^2^, Gründahl, M. ^2^, Unterecker, S.^3^, Kittel-Schneider, S.^2, 4, 5^, & Hein, G.^2^

## Model assumptions

For all models, we first tested the variance inflation factor (VIF) using the *performance* package (Lüdecke et al., 2021) in R. VIF values below 5 were considered indicative of low multicollinearity (see Tables S1, S3, S5, and S7). Next, we evaluated model assumptions for all models using simulated residuals generated with the *DHARMa* package (Hartig, 2016). Residuals were assessed for deviation from a uniform distribution using the Kolmogorov–Smirnov (KS) test. Significant deviations were detected for both the state anxiety and state social anxiety models (KS test, both *p* < .001; see Figures S1 and S2, Panels A). To address this, we applied a log(x + 1) transformation to these outcomes. Following transformation, the KS test no longer indicated significant deviation for the state anxiety model (see Figure S1, Panel B), and although the state social anxiety model continued to show deviation, visual inspection of residuals confirmed substantial improvement (see Figure S2, Panel B). Tests for under- or overdispersion indicated no significant dispersion in any model (all *p* ≥ .432). Heteroscedasticity was assessed using DHARMa’s quantile test, which indicated significant heteroscedasticity across all models (all *p* ≤ .006, see Figures S1-S4).

### State Anxiety Model

**Table S1.** VIFs for the state anxiety model

| Term | VIF | VIF_CI_low | VIF_CI_high | SE_factor |
| --- | --- | --- | --- | --- |
| group | 1.174 | 1.121 | 1.250 | 1.084 |
| SI_familiarity_cw | 1.270 | 1.207 | 1.352 | 1.127 |
| female | 1.073 | 1.035 | 1.152 | 1.036 |
| SI_gender_partner | 2.355 | 2.193 | 2.540 | 1.535 |
| type_of_day | 1.076 | 1.037 | 1.154 | 1.037 |
| SIAS_cg | 1.435 | 1.356 | 1.532 | 1.198 |
| ADS_cg | 2.482 | 2.308 | 2.679 | 1.576 |
| SI_count | 1.980 | 1.851 | 2.128 | 1.407 |
| SI_type_simple | 1.068 | 1.031 | 1.148 | 1.034 |
| SI_duration_cw | 1.147 | 1.097 | 1.221 | 1.071 |
| SI_caffeine | 1.035 | 1.009 | 1.145 | 1.018 |
| SI_nicotin | 1.043 | 1.013 | 1.140 | 1.021 |
| SI_alcohol | 1.042 | 1.013 | 1.140 | 1.021 |
| age_cb | 1.210 | 1.153 | 1.288 | 1.100 |
| SI_familiarity_cw:female | 1.197 | 1.142 | 1.274 | 1.094 |
| female_gender_partner | 1.327 | 1.259 | 1.414 | 1.152 |
| group:SI_familiarity_cw | 1.267 | 1.204 | 1.349 | 1.126 |
| group:female | 1.073 | 1.035 | 1.152 | 1.036 |
| group:SI_gender_partner | 1.267 | 1.204 | 1.349 | 1.126 |
| group:type_of_day | 1.075 | 1.037 | 1.153 | 1.037 |
| group:SIAS_cg | 1.414 | 1.337 | 1.509 | 1.189 |
| group:ADS_cg | 2.472 | 2.299 | 2.668 | 1.572 |
| group:SI_familiarity_cw:female | 1.191 | 1.137 | 1.268 | 1.091 |
| group:female:SI_gender_partner | 1.293 | 1.228 | 1.377 | 1.137 |

**
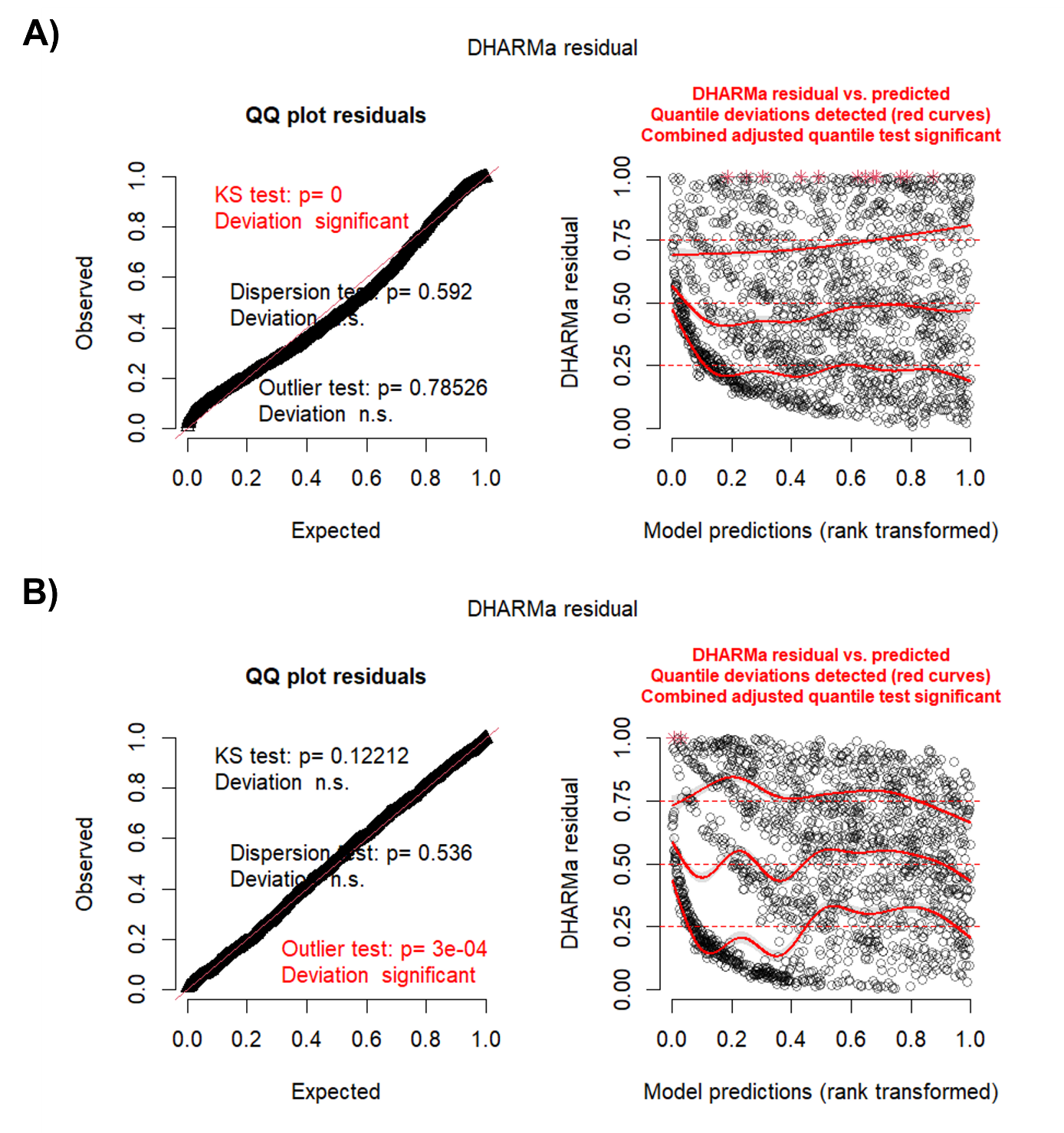
**

**Figure S1.** DHARMa residual plots for the state anxiety model with the untransformed outcome (Panel A) and the log(x + 1)-transformed outcome (Panel B).

### State Social Anxiety Model

**Table S2.** VIFs for the state social anxiety model

| Term | VIF | VIF_CI_low | VIF_CI_high | SE_factor |
| --- | --- | --- | --- | --- |
| group | 1.191 | 1.136 | 1.267 | 1.091 |
| SI_familiarity_cw | 1.265 | 1.203 | 1.347 | 1.125 |
| female | 1.077 | 1.038 | 1.155 | 1.038 |
| SI_gender_partner | 2.357 | 2.194 | 2.541 | 1.535 |
| type_of_day | 1.069 | 1.032 | 1.149 | 1.034 |
| SIAS_cg | 1.442 | 1.362 | 1.539 | 1.201 |
| ADS_cg | 2.495 | 2.320 | 2.693 | 1.580 |
| SI_count | 1.981 | 1.852 | 2.129 | 1.407 |
| SI_type_simple | 1.068 | 1.031 | 1.148 | 1.034 |
| SI_duration_cw | 1.144 | 1.095 | 1.219 | 1.070 |
| SI_caffeine | 1.036 | 1.009 | 1.144 | 1.018 |
| SI_nicotin | 1.045 | 1.015 | 1.139 | 1.022 |
| SI_alcohol | 1.041 | 1.012 | 1.140 | 1.020 |
| age_cb | 1.210 | 1.154 | 1.288 | 1.100 |
| SI_familiarity_cw:female | 1.197 | 1.141 | 1.274 | 1.094 |
| female:SI_gender_partner | 1.327 | 1.258 | 1.414 | 1.152 |
| group:SI_familiarity_cw | 1.262 | 1.200 | 1.344 | 1.123 |
| group:female | 1.076 | 1.038 | 1.154 | 1.037 |
| group:SI_gender_partner | 1.268 | 1.205 | 1.350 | 1.126 |
| group:type_of_day | 1.082 | 1.043 | 1.159 | 1.040 |
| group:SIAS_cg | 1.421 | 1.343 | 1.516 | 1.192 |
| group:ADS_cg | 2.485 | 2.311 | 2.683 | 1.577 |
| group:SI_familiarity_cw:female | 1.191 | 1.136 | 1.267 | 1.091 |
| group:female:SI_gender_partner | 1.293 | 1.228 | 1.377 | 1.137 |

**
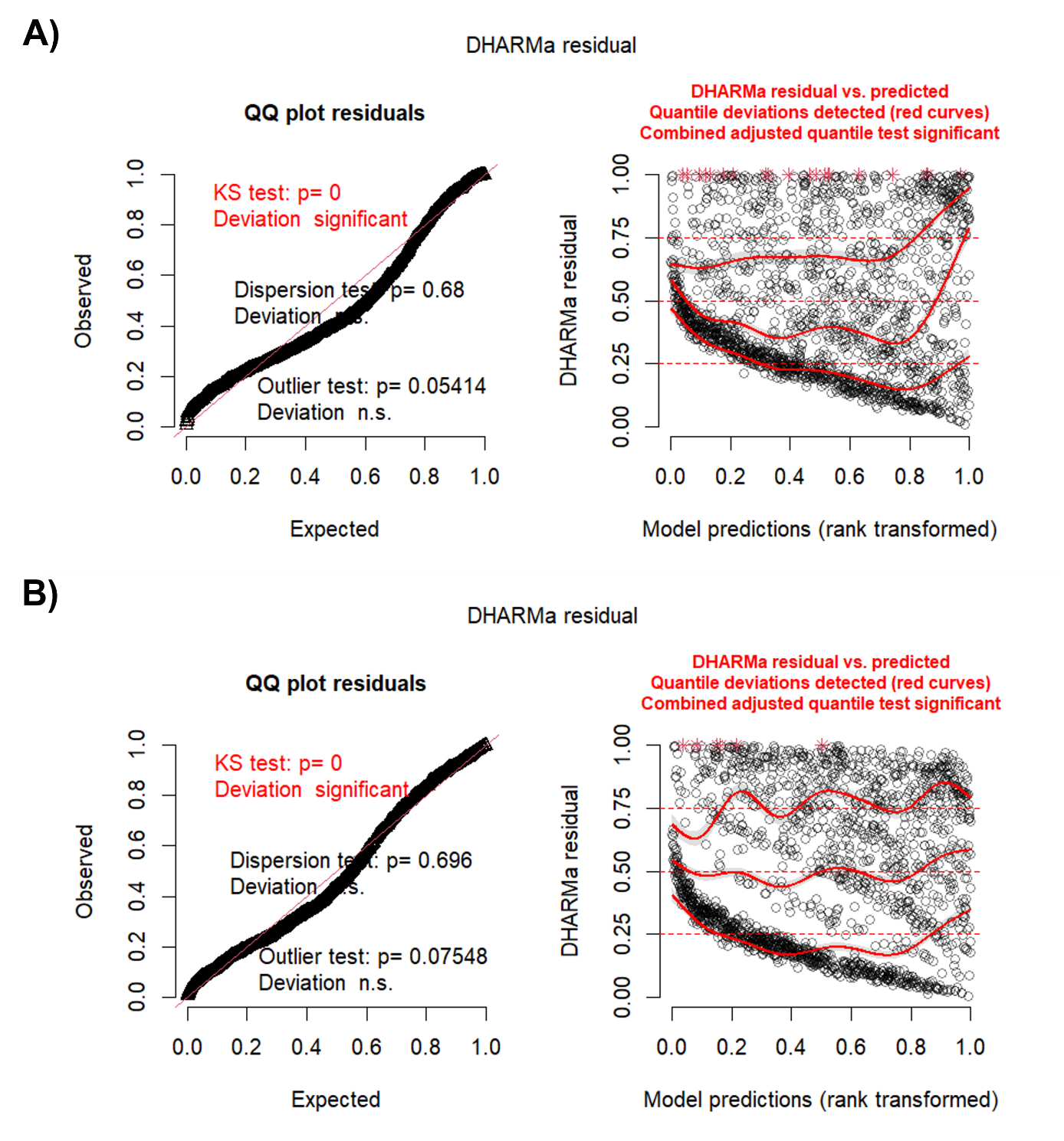
**

**Figure S2.** DHARMa **r**esidual plots for the state social anxiety model with the untransformed outcome (Panel A) and the log(x + 1)-transformed outcome (Panel B).

### HR Model

**Table S3.** VIFs for the HR model

| Term | VIF | VIF_CI_low | VIF_CI_high | SE_factor |
| --- | --- | --- | --- | --- |
| group | 1.051 | 1.011 | 1.242 | 1.025 |
| SI_familiarity_cw | 1.241 | 1.155 | 1.376 | 1.114 |
| female | 1.041 | 1.006 | 1.277 | 1.020 |
| SI_gender_partner | 1.321 | 1.222 | 1.465 | 1.150 |
| type_of_day | 1.084 | 1.031 | 1.229 | 1.041 |
| SI_caffeine | 1.043 | 1.007 | 1.268 | 1.021 |
| SI_nicotin | 1.067 | 1.019 | 1.228 | 1.033 |
| SI_alcohol | 1.039 | 1.005 | 1.287 | 1.019 |
| accel_EMA_cw | 1.105 | 1.046 | 1.241 | 1.051 |
| HR_BL_cg | 1.139 | 1.071 | 1.270 | 1.067 |
| SI_familiarity_cw:female | 1.207 | 1.126 | 1.339 | 1.099 |
| female:SI_gender_partner | 1.322 | 1.223 | 1.465 | 1.150 |
| group:SI_familiarity_cw | 1.255 | 1.166 | 1.391 | 1.120 |
| group:female | 1.036 | 1.004 | 1.306 | 1.018 |
| group:SI_gender_partner | 1.340 | 1.238 | 1.486 | 1.158 |
| group:type_of_day | 1.092 | 1.036 | 1.233 | 1.045 |
| group:SI_familiarity_cw:female | 1.201 | 1.121 | 1.333 | 1.096 |
| group:female:SI_gender_partner | 1.325 | 1.225 | 1.469 | 1.151 |

**
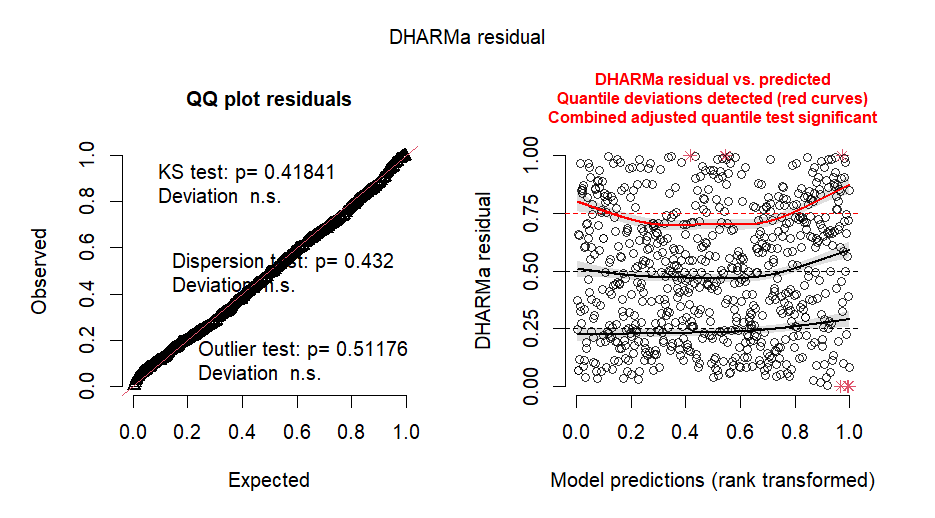
**

**Figure S3.** DHARMa **r**esidual plots for the HR model.

### HRV Model

**Table S4.** VIFs for the HRV model

| Term | VIF | VIF_CI_low | VIF_CI_high | SE_factor |
| --- | --- | --- | --- | --- |
| group | 1.154 | 1.083 | 1.286 | 1.074 |
| SI_familiarity_cw | 1.239 | 1.152 | 1.375 | 1.113 |
| female | 1.034 | 1.003 | 1.335 | 1.017 |
| SI_gender_partner | 1.316 | 1.217 | 1.461 | 1.147 |
| type_of_day | 1.088 | 1.033 | 1.233 | 1.043 |
| SI_caffeine | 1.037 | 1.005 | 1.305 | 1.018 |
| SI_nicotin | 1.058 | 1.014 | 1.236 | 1.029 |
| SI_alcohol | 1.034 | 1.003 | 1.339 | 1.017 |
| accel_EMA_cw | 1.051 | 1.010 | 1.248 | 1.025 |
| ln_RMSSD_BL_cg | 1.062 | 1.017 | 1.233 | 1.031 |
| SI_familiarity_cw:female | 1.214 | 1.132 | 1.348 | 1.102 |
| female:SI_gender_partner | 1.327 | 1.226 | 1.473 | 1.152 |
| group:SI_familiarity_cw | 1.257 | 1.167 | 1.395 | 1.121 |
| group:female | 1.033 | 1.003 | 1.354 | 1.016 |
| group:SI_gender_partner | 1.335 | 1.233 | 1.482 | 1.155 |
| group:type_of_day | 1.224 | 1.140 | 1.359 | 1.106 |
| group:SI_familiarity_cw:female | 1.207 | 1.126 | 1.341 | 1.099 |
| group:female:SI_gender_partner | 1.335 | 1.233 | 1.482 | 1.156 |


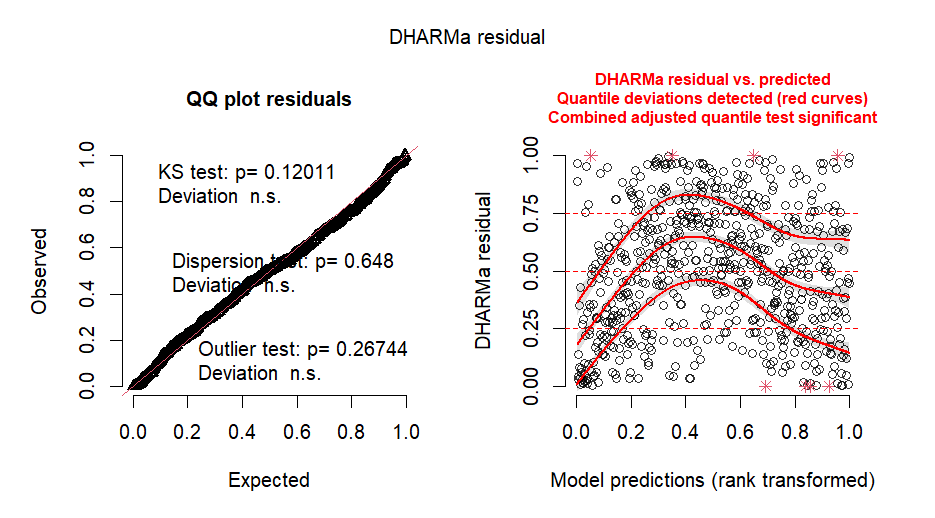


**Figure S4.** DHARMa residual plots for the HRV model.

## Fixed Effects

To address heteroscedasticity, we analyzed cluster-robust standard errors calculated with the CR2 method (Bell & McCaffrey, 2002), implemented in the *clubSandwich* package (Pustejovsky, 2016). For all fixed effects and a comparison between model-based and robust SEs (computed with the *CR2* package, Huang & Zhang, 2022) see Tables S5-S8.

**Table S5. Fixed effects of the state anxiety model**

| term | estimate | mb.se | robust.se | t.val | df | p.val | p.adj |
| --- | --- | --- | --- | --- | --- | --- | --- |
| (Intercept) | **0.894** | **0.053** | **0.042** | **21.243** | **69.354** | **0.000** | **0.000** |
| group1 | **0.532** | **0.076** | **0.078** | **6.771** | **83.530** | **0.000** | **0.000** |
| SI_familiarity_cw | **-0.039** | **0.005** | **0.008** | **-5.074** | **69.205** | **0.000** | **0.000** |
| female1 | 0.137 | 0.072 | 0.071 | 1.937 | 88.586 | 0.056 | 0.224 |
| SI_gender_partnerfemale_minus_male | -0.013 | 0.030 | 0.030 | -0.427 | 71.516 | 0.671 | 1.000 |
| SI_gender_partnerMixed_minus_notMixed | 0.106 | 0.036 | 0.047 | 2.279 | 75.438 | 0.026 | 0.104 |
| type_of_day1 | 0.039 | 0.031 | 0.032 | 1.228 | 101.625 | 0.222 | 0.888 |
| SIAS_cg | **0.012** | **0.003** | **0.003** | **4.098** | **37.660** | **0.000** | **0.000** |
| ADS_cg | **0.025** | **0.007** | **0.006** | **4.278** | **19.387** | **0.000** | **0.000** |
| SI_count2 | 0.048 | 0.032 | 0.034 | 1.401 | 75.285 | 0.165 | 0.660 |
| SI_type_simple2 | 0.016 | 0.033 | 0.034 | 0.456 | 67.885 | 0.650 | 1.000 |
| SI_duration_cw | 0.000 | 0.000 | 0.000 | 0.716 | 85.393 | 0.476 | 1.000 |
| SI_caffeine1 | -0.010 | 0.033 | 0.032 | -0.317 | 58.624 | 0.752 | 1.000 |
| SI_nicotin1 | -0.004 | 0.051 | 0.057 | -0.063 | 21.534 | 0.950 | 1.000 |
| SI_alcohol1 | **0.140** | **0.061** | **0.044** | **3.156** | **24.237** | **0.004** | **0.016** |
| age_cb | 0.004 | 0.003 | 0.004 | 0.880 | 25.798 | 0.387 | 1.000 |
| SI_familiarity_cw:female1 | -0.007 | 0.010 | 0.015 | -0.513 | 68.847 | 0.609 | 1.000 |
| female1:SI_gender_partnerfemale_minus_male | 0.094 | 0.062 | 0.060 | 1.562 | 72.049 | 0.123 | 0.492 |
| female1:SI_gender_partnerMixed_minus_notMixed | 0.028 | 0.054 | 0.079 | 0.354 | 67.553 | 0.725 | 1.000 |
| group1:SI_familiarity_cw | 0.008 | 0.011 | 0.015 | 0.537 | 69.407 | 0.593 | 1.000 |
| group1:female1 | -0.154 | 0.144 | 0.143 | -1.075 | 88.712 | 0.285 | 1.000 |
| group1:SI_gender_partnerfemale_minus_male | 0.099 | 0.061 | 0.060 | 1.643 | 71.928 | 0.105 | 0.420 |
| group1:SI_gender_partnerMixed_minus_notMixed | **-0.276** | **0.053** | **0.080** | **-3.448** | **66.928** | **0.001** | **0.004** |
| group1:type_of_day1 | 0.113 | 0.063 | 0.065 | 1.749 | 101.351 | 0.083 | 0.332 |
| group1:SIAS_cg | -0.002 | 0.006 | 0.006 | -0.295 | 37.346 | 0.769 | 1.000 |
| group1:ADS_cg | 0.012 | 0.015 | 0.012 | 1.020 | 19.538 | 0.320 | 1.000 |
| group1:SI_familiarity_cw:female1 | 0.020 | 0.021 | 0.029 | 0.696 | 68.519 | 0.489 | 1.000 |
| group1:female1:SI_gender_partnerfemale_minus_male | -0.271 | 0.123 | 0.118 | -2.304 | 71.027 | 0.024 | 0.096 |
| group1:female1:SI_gender_partnerMixed_minus_notMixed | 0.161 | 0.107 | 0.159 | 1.011 | 66.729 | 0.316 | 1.000 |

Note. mb.se = model based standard errors, robust.se = robust standard errors from CR2 method, t.val = t-value, df = degrees of freedom, p.val = unadjusted p-value, p.adj = Bonferroni adjusted p-value for 4 tests. Significant parameters after adjustment in bold.

**Table S6. Fixed effects of the state social anxiety model**

| term | estimate | mb.se | robust.se | t.val | df | p.val | p.adj |
| --- | --- | --- | --- | --- | --- | --- | --- |
| (Intercept) | **0.483** | **0.061** | **0.062** | **7.851** | **65.807** | **0.000** | **0.000** |
| group1 | 0.056 | 0.084 | 0.084 | 0.664 | 83.147 | 0.508 | 1.000 |
| SI_familiarity_cw | -0.014 | 0.006 | 0.012 | -1.146 | 69.060 | 0.256 | 1.000 |
| female1 | 0.134 | 0.079 | 0.078 | 1.703 | 88.434 | 0.092 | 0.368 |
| SI_gender_partnerfemale_minus_male | **0.162** | **0.037** | **0.044** | **3.642** | **71.665** | **0.001** | **0.004** |
| SI_gender_partnerMixed_minus_notMixed | 0.007 | 0.044 | 0.056 | 0.123 | 75.486 | 0.903 | 1.000 |
| type_of_day1 | **0.117** | **0.040** | **0.038** | **3.103** | **102.983** | **0.002** | **0.008** |
| SIAS_cg | **0.014** | **0.003** | **0.003** | **4.401** | **37.811** | **0.000** | **0.000** |
| ADS_cg | 0.016 | 0.008 | 0.009 | 1.793 | 19.441 | 0.089 | 0.356 |
| SI_count2 | **0.365** | **0.038** | **0.056** | **6.549** | **75.187** | **0.000** | **0.000** |
| SI_type_simple2 | 0.022 | 0.039 | 0.059 | 0.380 | 67.877 | 0.705 | 1.000 |
| SI_duration_cw | 0.000 | 0.000 | 0.000 | 1.407 | 85.333 | 0.163 | 0.652 |
| SI_caffeine1 | 0.007 | 0.039 | 0.038 | 0.184 | 58.933 | 0.855 | 1.000 |
| SI_nicotin1 | 0.009 | 0.061 | 0.051 | 0.186 | 22.101 | 0.854 | 1.000 |
| SI_alcohol1 | 0.109 | 0.074 | 0.086 | 1.266 | 24.189 | 0.218 | 0.872 |
| age_cb | 0.002 | 0.004 | 0.003 | 0.565 | 25.875 | 0.577 | 1.000 |
| SI_familiarity_cw:female1 | -0.003 | 0.013 | 0.024 | -0.122 | 68.835 | 0.903 | 1.000 |
| female1:SI_gender_partnerfemale_minus_male | -0.026 | 0.075 | 0.092 | -0.279 | 72.202 | 0.781 | 1.000 |
| female1:SI_gender_partnerMixed_minus_notMixed | -0.108 | 0.064 | 0.092 | -1.181 | 67.769 | 0.242 | 0.968 |
| group1:SI_familiarity_cw | **-0.068** | **0.013** | **0.024** | **-2.824** | **69.218** | **0.006** | **0.024** |
| group1:female1 | -0.154 | 0.158 | 0.159 | -0.969 | 88.507 | 0.335 | 1.000 |
| group1:SI_gender_partnerfemale_minus_male | -0.197 | 0.073 | 0.089 | -2.199 | 72.077 | 0.031 | 0.124 |
| group1:SI_gender_partnerMixed_minus_notMixed | **-0.320** | **0.064** | **0.093** | **-3.433** | **67.093** | **0.001** | **0.004** |
| group1:type_of_day1 | -0.041 | 0.081 | 0.074 | -0.558 | 102.739 | 0.578 | 1.000 |
| group1:SIAS_cg | 0.000 | 0.006 | 0.006 | 0.014 | 37.571 | 0.989 | 1.000 |
| group1:ADS_cg | 0.017 | 0.016 | 0.018 | 0.934 | 19.601 | 0.362 | 1.000 |
| group1:SI_familiarity_cw:female1 | -0.015 | 0.025 | 0.049 | -0.305 | 68.504 | 0.761 | 1.000 |
| group1:female1:SI_gender_partnerfemale_minus_male | -0.426 | 0.147 | 0.179 | -2.377 | 71.190 | 0.020 | 0.080 |
| group1:female1:SI_gender_partnerMixed_minus_notMixed | 0.165 | 0.128 | 0.185 | 0.895 | 66.941 | 0.374 | 1.000 |

Note. mb.se = model based standard errors, robust.se = robust standard errors from CR2 method, t.val = t-value, df = degrees of freedom, p.val = unadjusted p-value, p.adj = Bonferroni adjusted p-value for 4 tests. Significant parameters after adjustment in bold.

**Table S7. Fixed effects of the HR model**

| term | estimate | mb.se | robust.se | t.val | df | p.val | p.adj |
| --- | --- | --- | --- | --- | --- | --- | --- |
| (Intercept) | **92.725** | **1.398** | **1.394** | **66.531** | **23.625** | **0.000** | **0.000** |
| group1 | **-11.075** | **1.914** | **1.820** | **-6.085** | **42.248** | **0.000** | **0.000** |
| SI_familiarity_cw | -0.150 | 0.150 | 0.128 | -1.170 | 28.317 | 0.252 | 1.000 |
| female1 | -1.905 | 1.903 | 1.916 | -0.994 | 42.484 | 0.326 | 1.000 |
| SI_gender_partnerfemale_minus_male | 0.025 | 0.877 | 0.686 | 0.036 | 23.616 | 0.972 | 1.000 |
| SI_gender_partnerMixed_minus_notMixed | **2.795** | **0.762** | **0.753** | **3.710** | **28.066** | **0.001** | **0.004** |
| type_of_day1 | -0.483 | 0.926 | 0.912 | -0.529 | 40.363 | 0.599 | 1.000 |
| SI_caffeine1 | -0.179 | 0.854 | 0.843 | -0.213 | 24.203 | 0.833 | 1.000 |
| SI_nicotin1 | -3.806 | 1.506 | 1.212 | -3.141 | 6.928 | 0.017 | 0.068 |
| SI_alcohol1 | -5.313 | 1.730 | 1.807 | -2.940 | 8.904 | 0.017 | 0.068 |
| accel_EMA_cw | **114.961** | **6.406** | **15.397** | **7.466** | **23.059** | **0.000** | **0.000** |
| HR_BL_cg | **0.458** | **0.034** | **0.055** | **8.317** | **20.456** | **0.000** | **0.000** |
| SI_familiarity_cw:female1 | 0.270 | 0.295 | 0.246 | 1.098 | 27.587 | 0.282 | 1.000 |
| female1:SI_gender_partnerfemale_minus_male | -1.282 | 1.754 | 1.412 | -0.907 | 23.527 | 0.373 | 1.000 |
| female1:SI_gender_partnerMixed_minus_notMixed | 0.200 | 1.513 | 1.584 | 0.126 | 27.768 | 0.900 | 1.000 |
| group1:SI_familiarity_cw | -0.115 | 0.301 | 0.249 | -0.460 | 28.299 | 0.649 | 1.000 |
| group1:female1 | 0.564 | 3.797 | 3.744 | 0.151 | 42.649 | 0.881 | 1.000 |
| group1:SI_gender_partnerfemale_minus_male | -2.011 | 1.759 | 1.497 | -1.344 | 23.449 | 0.192 | 0.768 |
| group1:SI_gender_partnerMixed_minus_notMixed | 0.339 | 1.521 | 1.475 | 0.230 | 27.912 | 0.820 | 1.000 |
| group1:type_of_day1 | 0.346 | 1.853 | 1.804 | 0.192 | 40.313 | 0.849 | 1.000 |
| group1:SI_familiarity_cw:female1 | 0.231 | 0.589 | 0.486 | 0.477 | 27.163 | 0.638 | 1.000 |
| group1:female1:SI_gender_partnerfemale_minus_male | **-8.885** | **3.514** | **2.918** | **-3.044** | **23.582** | **0.006** | **0.024** |
| group1:female1:SI_gender_partnerMixed_minus_notMixed | 0.988 | 3.031 | 3.076 | 0.321 | 27.685 | 0.750 | 1.000 |

Note. mb.se = model based standard errors, robust.se = robust standard errors from CR2 method, t.val = t-value, df = degrees of freedom, p.val = unadjusted p-value, p.adj = Bonferroni adjusted p-value for 4 tests. Significant parameters after adjustment in bold.

**Table S8. Fixed effects of the HRV model**

| term | estimate | mb.se | robust.se | t.val | df | p.val | p.adj |
| --- | --- | --- | --- | --- | --- | --- | --- |
| (Intercept) | **3.047** | **0.074** | **0.073** | **41.527** | **28.121** | **0.000** | **0.000** |
| group1 | **0.782** | **0.112** | **0.106** | **7.353** | **42.880** | **0.000** | **0.000** |
| SI_familiarity_cw | 0.002 | 0.007 | 0.008 | 0.207 | 27.837 | 0.838 | 1.000 |
| female1 | -0.000 | 0.107 | 0.105 | -0.003 | 43.018 | 0.997 | 1.000 |
| SI_gender_partnerfemale_minus_male | -0.009 | 0.041 | 0.044 | -0.211 | 23.361 | 0.835 | 1.000 |
| SI_gender_partnerMixed_minus_notMixed | -0.088 | 0.036 | 0.034 | -2.598 | 26.527 | 0.015 | 0.060 |
| type_of_day1 | 0.044 | 0.046 | 0.047 | 0.937 | 37.622 | 0.355 | 1.000 |
| SI_caffeine1 | 0.000 | 0.041 | 0.053 | 0.006 | 23.196 | 0.995 | 1.000 |
| SI_nicotin1 | -0.024 | 0.072 | 0.088 | -0.276 | 6.593 | 0.791 | 1.000 |
| SI_alcohol1 | 0.082 | 0.081 | 0.082 | 0.995 | 8.873 | 0.346 | 1.000 |
| accel_EMA_cw | **-2.667** | **0.294** | **0.435** | **-6.129** | **23.527** | **0.000** | **0.000** |
| ln_RMSSD_BL_cg | **0.538** | **0.035** | **0.054** | **9.894** | **15.357** | **0.000** | **0.000** |
| SI_familiarity_cw:female1 | -0.023 | 0.014 | 0.017 | -1.351 | 27.119 | 0.188 | 0.752 |
| female1:SI_gender_partnerfemale_minus_male | 0.088 | 0.083 | 0.089 | 0.981 | 23.313 | 0.337 | 1.000 |
| female1:SI_gender_partnerMixed_minus_notMixed | -0.050 | 0.072 | 0.070 | -0.721 | 25.952 | 0.477 | 1.000 |
| group1:SI_familiarity_cw | -0.018 | 0.014 | 0.016 | -1.089 | 27.839 | 0.285 | 1.000 |
| group1:female1 | -0.205 | 0.214 | 0.207 | -0.989 | 42.952 | 0.328 | 1.000 |
| group1:SI_gender_partnerfemale_minus_male | 0.032 | 0.083 | 0.091 | 0.349 | 23.222 | 0.730 | 1.000 |
| group1:SI_gender_partnerMixed_minus_notMixed | 0.018 | 0.073 | 0.066 | 0.269 | 26.578 | 0.790 | 1.000 |
| group1:type_of_day1 | -0.038 | 0.092 | 0.094 | -0.407 | 37.575 | 0.686 | 1.000 |
| group1:SI_familiarity_cw:female1 | -0.035 | 0.028 | 0.032 | -1.094 | 26.771 | 0.284 | 1.000 |
| group1:female1:SI_gender_partnerfemale_minus_male | 0.302 | 0.166 | 0.182 | 1.659 | 23.338 | 0.110 | 0.440 |
| group1:female1:SI_gender_partnerMixed_minus_notMixed | 0.032 | 0.145 | 0.143 | 0.227 | 25.812 | 0.823 | 1.000 |

Note. mb.se = model based standard errors, robust.se = robust standard errors from CR2 method, t.val = t-value, df = degrees of freedom, p.val = unadjusted p-value, p.adj = Bonferroni adjusted p-value for 4 tests. Significant parameters after adjustment in bold.

## Exploratory Analyses of Social Interaction Pleasantness and Partner Familiarity

To address a reviewer suggestion, we conducted additional exploratory mixed-effects analyses examining social interaction pleasantness, its interaction with partner familiarity, and the three-way interaction with group (patients vs. controls). These analyses were conducted separately for self-report outcomes (state anxiety, state social interaction anxiety) and physiological outcomes (heart rate, heart rate variability).

### State Anxiety Model

Including social interaction pleasantness, its interaction with partner familiarity, and the three-way interaction with group resulted in a significantly improved model fit compared to the final main-text model, χ²(3) = 118.65, *p* < .001. Within this model, pleasantness showed a significant main effect on state anxiety, *B* = −0.084, *p* < .001, whereas neither the pleasantness × familiarity interaction nor the three-way interaction with group was statistically significant. All fixed effects are depicted in Table S9.

### State Social Anxiety Model

Including social interaction pleasantness, its interaction with partner familiarity, and the three-way interaction with group resulted in a significantly improved model fit compared to the final main-text model, χ²(3) = 76.247, *p* < .001. Within this model, pleasantness showed a significant main effect on state social anxiety, *B* = −0.081, *p* < .001, whereas neither the pleasantness × familiarity interaction nor the three-way interaction with group was statistically significant. All fixed effects are depicted in Table S10.

### HR Model

Including social interaction pleasantness, its interaction with partner familiarity, and the three-way interaction with group did not improve model fit compared to the final main-text model, χ²(3) = 2.53, *p* = .469. Pleasantness did not show a main effect on heart rate, and no interaction effects were supported. Full fixed-effect estimates for this model are reported in Table S11.

### HRV Model

Including social interaction pleasantness, its interaction with partner familiarity, and the three-way interaction with group did not improve model fit compared to the final main-text model, χ²(3) = 0.08, *p* = .994. Pleasantness did not show a main effect on heart rate variability, and no interaction effects were supported. Full fixed-effect estimates for this model are reported in Table S12.

**Table S9. Fixed effects of the state anxiety model including social interaction pleasantness**

| term | estimate | mb.se | robust.se | t.val | df | p.val | p.adj |
| --- | --- | --- | --- | --- | --- | --- | --- |
| **(Intercept)** | **0.917** | **0.052** | **0.043** | **21.215** | **71.598** | **0.000** | **0.000** |
| **group1** | **0.545** | **0.076** | **0.076** | **7.199** | **83.982** | **0.000** | **0.000** |
| SI_familiarity_cw | -0.018 | 0.006 | 0.009 | -2.061 | 62.974 | 0.043 | 0.172 |
| female1 | 0.133 | 0.072 | 0.071 | 1.881 | 88.633 | 0.063 | 0.252 |
| SI_gender_partnermale_minus_male | -0.019 | 0.029 | 0.029 | -0.648 | 71.773 | 0.519 | 1.000 |
| SI_gender_partnerMixed_minus_notMixed | 0.089 | 0.035 | 0.044 | 2.006 | 75.182 | 0.048 | 0.192 |
| type_of_day1 | 0.047 | 0.030 | 0.031 | 1.517 | 101.702 | 0.132 | 0.528 |
| **SIAS_cg** | **0.012** | **0.003** | **0.003** | **4.064** | **37.594** | **0.000** | **0.000** |
| **ADS_cg** | **0.025** | **0.007** | **0.006** | **4.327** | **19.383** | **0.000** | **0.000** |
| SI_count2 | 0.037 | 0.030 | 0.032 | 1.153 | 75.718 | 0.252 | 1.000 |
| SI_type_simple2 | 0.009 | 0.032 | 0.031 | 0.282 | 67.692 | 0.779 | 1.000 |
| SI_duration_cw | 0.000 | 0.000 | 0.000 | 2.196 | 84.904 | 0.031 | 0.124 |
| SI_caffeine1 | 0.000 | 0.031 | 0.029 | 0.000 | 58.421 | 1.000 | 1.000 |
| SI_nicotin1 | -0.013 | 0.050 | 0.053 | -0.240 | 21.359 | 0.813 | 1.000 |
| SI_alcohol1 | 0.091 | 0.059 | 0.041 | 2.191 | 24.515 | 0.038 | 0.152 |
| age_cb | 0.004 | 0.003 | 0.004 | 0.971 | 25.816 | 0.340 | 1.000 |
| **SI_pleasantness_cw** | **-0.084** | **0.008** | **0.010** | **-8.246** | **70.523** | **0.000** | **0.000** |
| SI_familiarity_cw:female1 | -0.008 | 0.010 | 0.015 | -0.575 | 68.160 | 0.567 | 1.000 |
| female1:SI_gender_partnermale_minus_male | 0.076 | 0.060 | 0.059 | 1.289 | 71.432 | 0.201 | 0.804 |
| female1:SI_gender_partnerMixed_minus_notMixed | 0.054 | 0.052 | 0.075 | 0.729 | 67.473 | 0.468 | 1.000 |
| group1:SI_familiarity_cw | 0.014 | 0.011 | 0.015 | 0.943 | 59.274 | 0.350 | 1.000 |
| group1:female1 | -0.148 | 0.145 | 0.143 | -1.034 | 88.817 | 0.304 | 1.000 |
| group1:SI_gender_partnermale_minus_male | 0.076 | 0.059 | 0.058 | 1.312 | 72.207 | 0.194 | 0.776 |
| **group1:SI_gender_partnerMixed_minus_notMixed** | **-0.251** | **0.052** | **0.075** | **-3.326** | **66.464** | **0.001** | **0.004** |
| group1:type_of_day1 | 0.092 | 0.061 | 0.063 | 1.463 | 101.450 | 0.147 | 0.588 |
| group1:SIAS_cg | -0.001 | 0.006 | 0.006 | -0.222 | 37.259 | 0.825 | 1.000 |
| group1:ADS_cg | 0.011 | 0.015 | 0.012 | 0.948 | 19.531 | 0.355 | 1.000 |
| SI_familiarity_cw:SI_pleasantness_cw | 0.002 | 0.003 | 0.003 | 0.500 | 43.405 | 0.620 | 1.000 |
| group1:SI_familiarity_cw:female1 | 0.025 | 0.020 | 0.029 | 0.857 | 68.143 | 0.394 | 1.000 |
| group1:female1:SI_gender_partnermale_minus_male | -0.238 | 0.119 | 0.115 | -2.074 | 70.515 | 0.042 | 0.168 |
| group1:female1:SI_gender_partnerMixed_minus_notMixed | 0.132 | 0.103 | 0.150 | 0.879 | 66.484 | 0.383 | 1.000 |
| group1:SI_familiarity_cw:SI_pleasantness_cw | -0.003 | 0.007 | 0.007 | -0.395 | 41.859 | 0.695 | 1.000 |

Note. mb.se = model based standard errors, robust.se = robust standard errors from CR2 method, t.val = t-value, df = degrees of freedom, p.val = unadjusted p-value, p.adj = Bonferroni adjusted p-value for 4 tests. Significant parameters after adjustment in bold.

**Table S10. Fixed effects of the state social anxiety model including social interaction pleasantness**

| term | estimate | mb.se | robust.se | t.val | df | p.val | p.adj |
| --- | --- | --- | --- | --- | --- | --- | --- |
| **(Intercept)** | **0.500** | **0.060** | **0.062** | **8.122** | **67.611** | **0.000** | **0.000** |
| group1 | 0.042 | 0.084 | 0.085 | 0.492 | 83.976 | 0.624 | 1.000 |
| SI_familiarity_cw | 0.011 | 0.007 | 0.012 | 0.922 | 62.793 | 0.360 | 1.000 |
| female1 | 0.131 | 0.079 | 0.079 | 1.664 | 88.481 | 0.100 | 0.400 |
| **SI_gender_partnermale_minus_male** | **0.156** | **0.036** | **0.045** | **3.438** | **71.903** | **0.001** | **0.004** |
| SI_gender_partnerMixed_minus_notMixed | -0.010 | 0.043 | 0.053 | -0.198 | 75.218 | 0.843 | 1.000 |
| **type_of_day1** | **0.125** | **0.040** | **0.037** | **3.354** | **103.174** | **0.001** | **0.004** |
| **SIAS_cg** | **0.013** | **0.003** | **0.003** | **4.330** | **37.782** | **0.000** | **0.000** |
| ADS_cg | 0.017 | 0.008 | 0.009 | 1.847 | 19.427 | 0.080 | 0.320 |
| **SI_count2** | **0.356** | **0.037** | **0.052** | **6.870** | **75.612** | **0.000** | **0.000** |
| SI_type_simple2 | 0.013 | 0.039 | 0.054 | 0.236 | 67.652 | 0.814 | 1.000 |
| SI_duration_cw | 0.000 | 0.000 | 0.000 | 2.262 | 84.843 | 0.026 | 0.104 |
| SI_caffeine1 | 0.022 | 0.038 | 0.037 | 0.594 | 58.765 | 0.555 | 1.000 |
| SI_nicotin1 | 0.006 | 0.060 | 0.056 | 0.114 | 22.008 | 0.910 | 1.000 |
| SI_alcohol1 | 0.064 | 0.072 | 0.085 | 0.753 | 24.448 | 0.458 | 1.000 |
| age_cb | 0.002 | 0.004 | 0.003 | 0.571 | 25.897 | 0.573 | 1.000 |
| **SI_pleasantness_cw** | **-0.081** | **0.009** | **0.012** | **-6.637** | **70.273** | **0.000** | **0.000** |
| SI_familiarity_cw:female1 | -0.003 | 0.012 | 0.024 | -0.147 | 68.092 | 0.883 | 1.000 |
| female1:SI_gender_partnermale_minus_male | -0.051 | 0.073 | 0.093 | -0.552 | 71.549 | 0.583 | 1.000 |
| female1:SI_gender_partnerMixed_minus_notMixed | -0.081 | 0.063 | 0.087 | -0.936 | 67.703 | 0.353 | 1.000 |
| group1:SI_familiarity_cw | -0.057 | 0.013 | 0.024 | -2.403 | 59.035 | 0.019 | 0.076 |
| group1:female1 | -0.142 | 0.158 | 0.159 | -0.890 | 88.602 | 0.376 | 1.000 |
| group1:SI_gender_partnermale_minus_male | -0.211 | 0.072 | 0.092 | -2.307 | 72.337 | 0.024 | 0.096 |
| **group1:SI_gender_partnerMixed_minus_notMixed** | **-0.298** | **0.063** | **0.089** | **-3.355** | **66.655** | **0.001** | **0.004** |
| group1:type_of_day1 | -0.057 | 0.080 | 0.072 | -0.791 | 102.956 | 0.431 | 1.000 |
| group1:SIAS_cg | 0.000 | 0.006 | 0.006 | 0.069 | 37.525 | 0.945 | 1.000 |
| group1:ADS_cg | 0.017 | 0.016 | 0.018 | 0.932 | 19.585 | 0.363 | 1.000 |
| SI_familiarity_cw:SI_pleasantness_cw | 0.004 | 0.004 | 0.004 | 0.864 | 43.369 | 0.392 | 1.000 |
| group1:SI_familiarity_cw:female1 | -0.007 | 0.025 | 0.047 | -0.154 | 68.095 | 0.878 | 1.000 |
| group1:female1:SI_gender_partnermale_minus_male | -0.385 | 0.144 | 0.180 | -2.136 | 70.638 | 0.036 | 0.144 |
| group1:female1:SI_gender_partnerMixed_minus_notMixed | 0.127 | 0.126 | 0.175 | 0.727 | 66.713 | 0.470 | 1.000 |
| group1:SI_familiarity_cw:SI_pleasantness_cw | 0.017 | 0.008 | 0.009 | 1.916 | 41.806 | 0.062 | 0.248 |

Note. mb.se = model based standard errors, robust.se = robust standard errors from CR2 method, t.val = t-value, df = degrees of freedom, p.val = unadjusted p-value, p.adj = Bonferroni adjusted p-value for 4 tests. Significant parameters after adjustment in bold.

**Table S11. Fixed effects of the HR model including social interaction pleasantness**

| term | estimate | mb.se | robust.se | t.val | df | p.val | p.adj |
| --- | --- | --- | --- | --- | --- | --- | --- |
| **(Intercept)** | **92.553** | **1.411** | **1.453** | **63.701** | **24.013** | **0.000** | **0.000** |
| **group1** | **-11.419** | **1.934** | **1.891** | **-6.038** | **42.180** | **0.000** | **0.000** |
| SI_familiarity_cw | -0.066 | 0.171 | 0.177 | -0.372 | 27.837 | 0.713 | 1.000 |
| female1 | -1.927 | 1.908 | 1.932 | -0.998 | 42.507 | 0.324 | 1.000 |
| SI_gender_partnerfemale_minus_male | -0.048 | 0.882 | 0.687 | -0.070 | 23.386 | 0.945 | 1.000 |
| **SI_gender_partnerMixed_minus_notMixed** | **2.841** | **0.764** | **0.743** | **3.825** | **28.054** | **0.001** | **0.004** |
| type_of_day1 | -0.427 | 0.928 | 0.943 | -0.453 | 40.140 | 0.653 | 1.000 |
| SI_caffeine1 | -0.095 | 0.858 | 0.862 | -0.111 | 23.993 | 0.913 | 1.000 |
| SI_nicotin1 | -3.597 | 1.515 | 1.258 | -2.859 | 7.052 | 0.024 | 0.096 |
| SI_alcohol1 | -5.382 | 1.739 | 1.765 | -3.049 | 9.042 | 0.014 | 0.056 |
| **accel_EMA_cw** | **115.229** | **6.440** | **15.425** | **7.470** | **23.010** | **0.000** | **0.000** |
| **HR_BL_cg** | **0.460** | **0.034** | **0.057** | **8.101** | **20.658** | **0.000** | **0.000** |
| SI_pleasantness_cw | -0.028 | 0.224 | 0.268 | -0.106 | 32.742 | 0.916 | 1.000 |
| SI_familiarity_cw:female1 | 0.292 | 0.297 | 0.252 | 1.159 | 27.247 | 0.257 | 1.000 |
| female1:SI_gender_partnerfemale_minus_male | -1.183 | 1.761 | 1.416 | -0.836 | 23.722 | 0.412 | 1.000 |
| female1:SI_gender_partnerMixed_minus_notMixed | 0.127 | 1.531 | 1.634 | 0.078 | 27.798 | 0.938 | 1.000 |
| group1:SI_familiarity_cw | 0.038 | 0.318 | 0.289 | 0.132 | 26.007 | 0.896 | 1.000 |
| group1:female1 | 0.537 | 3.808 | 3.749 | 0.143 | 42.661 | 0.887 | 1.000 |
| group1:SI_gender_partnerfemale_minus_male | -2.209 | 1.766 | 1.487 | -1.486 | 23.685 | 0.151 | 0.604 |
| group1:SI_gender_partnerMixed_minus_notMixed | 0.392 | 1.522 | 1.468 | 0.267 | 27.928 | 0.792 | 1.000 |
| group1:type_of_day1 | 0.516 | 1.859 | 1.835 | 0.281 | 40.140 | 0.780 | 1.000 |
| SI_familiarity_cw:SI_pleasantness_cw | 0.079 | 0.090 | 0.099 | 0.797 | 20.683 | 0.435 | 1.000 |
| group1:SI_familiarity_cw:female1 | 0.317 | 0.593 | 0.494 | 0.643 | 27.081 | 0.526 | 1.000 |
| **group1:female1:SI_gender_partnerfemale_minus_male** | **-8.615** | **3.536** | **2.946** | **-2.924** | **24.046** | **0.007** | **0.028** |
| group1:female1:SI_gender_partnerMixed_minus_notMixed | 0.504 | 3.070 | 3.200 | 0.157 | 27.527 | 0.876 | 1.000 |
| group1:SI_familiarity_cw:SI_pleasantness_cw | 0.255 | 0.179 | 0.198 | 1.285 | 20.751 | 0.213 | 0.852 |

Note. mb.se = model based standard errors, robust.se = robust standard errors from CR2 method, t.val = t-value, df = degrees of freedom, p.val = unadjusted p-value, p.adj = Bonferroni adjusted p-value for 4 tests. Significant parameters after adjustment in bold.

**Table S12. Fixed effects of the HRV model including social interaction pleasantness**

| term | estimate | mb.se | robust.se | t.val | df | p.val | p.adj |
| --- | --- | --- | --- | --- | --- | --- | --- |
| **(Intercept)** | **3.049** | **0.074** | **0.076** | **40.345** | **28.333** | **0.000** | **0.000** |
| **group1** | **0.784** | **0.112** | **0.109** | **7.177** | **42.789** | **0.000** | **0.000** |
| SI_familiarity_cw | 0.002 | 0.008 | 0.010 | 0.158 | 27.503 | 0.876 | 1.000 |
| female1 | -0.000 | 0.107 | 0.105 | -0.004 | 43.010 | 0.996 | 1.000 |
| SI_gender_partnerfemale_minus_male | -0.008 | 0.042 | 0.045 | -0.183 | 23.117 | 0.856 | 1.000 |
| SI_gender_partnerMixed_minus_notMixed | -0.088 | 0.037 | 0.034 | -2.603 | 26.510 | 0.015 | 0.060 |
| type_of_day1 | 0.043 | 0.046 | 0.048 | 0.912 | 37.437 | 0.367 | 1.000 |
| SI_caffeine1 | 0.000 | 0.041 | 0.054 | 0.001 | 22.990 | 0.999 | 1.000 |
| SI_nicotin1 | -0.026 | 0.073 | 0.089 | -0.296 | 6.740 | 0.776 | 1.000 |
| SI_alcohol1 | 0.081 | 0.082 | 0.084 | 0.965 | 9.018 | 0.360 | 1.000 |
| **accel_EMA_cw** | **-2.664** | **0.297** | **0.432** | **-6.169** | **23.519** | **0.000** | **0.000** |
| **ln_RMSSD_BL_cg** | **0.539** | **0.035** | **0.054** | **9.946** | **15.569** | **0.000** | **0.000** |
| SI_pleasantness_cw | -0.001 | 0.011 | 0.011 | -0.111 | 32.648 | 0.913 | 1.000 |
| SI_familiarity_cw:female1 | -0.023 | 0.014 | 0.017 | -1.306 | 26.852 | 0.203 | 0.812 |
| female1:SI_gender_partnerfemale_minus_male | 0.088 | 0.083 | 0.091 | 0.966 | 23.608 | 0.344 | 1.000 |
| female1:SI_gender_partnerMixed_minus_notMixed | -0.048 | 0.073 | 0.073 | -0.660 | 25.773 | 0.515 | 1.000 |
| group1:SI_familiarity_cw | -0.019 | 0.015 | 0.017 | -1.125 | 25.830 | 0.271 | 1.000 |
| group1:female1 | -0.204 | 0.214 | 0.206 | -0.989 | 42.944 | 0.328 | 1.000 |
| group1:SI_gender_partnerfemale_minus_male | 0.033 | 0.084 | 0.091 | 0.367 | 23.483 | 0.717 | 1.000 |
| group1:SI_gender_partnerMixed_minus_notMixed | 0.017 | 0.073 | 0.066 | 0.257 | 26.597 | 0.799 | 1.000 |
| group1:type_of_day1 | -0.040 | 0.093 | 0.094 | -0.429 | 37.410 | 0.670 | 1.000 |
| SI_familiarity_cw:SI_pleasantness_cw | -0.001 | 0.004 | 0.004 | -0.256 | 20.426 | 0.801 | 1.000 |
| group1:SI_familiarity_cw:female1 | -0.036 | 0.028 | 0.034 | -1.063 | 26.752 | 0.297 | 1.000 |
| group1:female1:SI_gender_partnerfemale_minus_male | 0.298 | 0.167 | 0.185 | 1.607 | 23.918 | 0.121 | 0.484 |
| group1:female1:SI_gender_partnerMixed_minus_notMixed | 0.033 | 0.147 | 0.148 | 0.222 | 25.480 | 0.826 | 1.000 |
| group1:SI_familiarity_cw:SI_pleasantness_cw | -0.001 | 0.008 | 0.007 | -0.186 | 20.491 | 0.854 | 1.000 |

Note. mb.se = model based standard errors, robust.se = robust standard errors from CR2 method, t.val = t-value, df = degrees of freedom, p.val = unadjusted p-value, p.adj = Bonferroni adjusted p-value for 4 tests. Significant parameters after adjustment in bold.

## Simulation-based Sensitivity Analyses

Because statistical power in linear mixed-effects models depends on the hierarchical structure of the data and the associated variance components, we conducted simulation-based sensitivity analyses to evaluate the detectability of effects that were central to our research questions. Rather than assessing sensitivity for all fixed effects in each model, we focused on a targeted set of theoretically relevant main effects and interactions that directly correspond to our a priori hypotheses and primary conclusions. For the self-report outcomes, this included the main effect of interaction partner familiarity and its interaction with group. For the physiological outcomes, sensitivity analyses focused on key group effects and higher-order interactions that were central to the interpretation of the results.

All sensitivity analyses were conducted using the *simr* package and were based on the fitted mixed-effects models. For each effect of interest, the corresponding fixed-effect coefficient was systematically varied across a predefined grid of plausible effect sizes, while all other model parameters, including variance components and random-effects structure, were held constant. For each grid point, 500 datasets were simulated, and effects were tested using two-sided *t*-tests at α = .05. Statistical power was defined as the proportion of simulations in which the effect of interest reached statistical significance. Ninety-five percent confidence intervals for power were obtained from the simulation output.

**Self-Reported Measures**

*Main Effect of Familiarity*

To assess sensitivity for detecting familiarity-related effects independent of group differences, we first examined the main effect of interaction partner familiarity in the self-report models. The fixed-effect coefficient for familiarity was varied over a grid ranging from *B* = 0 to 0.05 in steps of 0.0025.

For state anxiety, the target power of 80% was reached at an effect size of approximately *B* = 0.025 (power = 82.2%, 95% CI [78.6%, 85.5%]). This indicates that the present design was sufficiently sensitive to detect small-to-moderate familiarity-related reductions in state anxiety.

For state social interaction anxiety, the corresponding threshold of 80% power was reached at approximately |*B|* = 0.03 (power = 81.6%, 95% CI [77.9%, 84.9%]). These findings indicate comparable sensitivity for detecting main effects of familiarity across both self-report outcomes.

*Group × Familiarity Interaction*

To assess sensitivity for detecting group differences in familiarity-related effects, we next evaluated the Group × Familiarity interaction, defined as the primary effect of interest for testing whether social buffering effects differ between patients with depression and healthy controls. Starting from the fitted model, the fixed-effect coefficient of this interaction was systematically varied across a grid of plausible effect sizes, while holding all other model parameters constant.

For state anxiety, the Group × Familiarity interaction reached the target power of 80% at an effect size of approximately |*B|* = 0.05 (power = 83.2%, 95% CI [79.6%, 86.4%]), indicating sufficient sensitivity to detect interaction effects of this magnitude under the present study design.

For state social interaction anxiety, the corresponding threshold was reached at approximately |*B|* = 0.06 (power = 83.0%, 95% CI [79.4%, 86.2%]), suggesting comparable sensitivity for detecting group differences in familiarity-related effects.

**Autonomic Outcomes**

*Main Effect of Group*

For HR, sensitivity for the main effect of group was evaluated by varying the fixed-effect coefficient over a grid from B = 0 to 8 in steps of 0.5. The target power of 80% was reached at approximately |*B*| = 7.0 (power = 83.2%, 95% CI [79.6%, 86.4%]). The observed group effect in the empirical analysis (B = −11.08) exceeded this threshold, indicating that the present design had sufficient sensitivity to detect the observed group difference in heart rate.

For HRV, sensitivity for the main effect of group was evaluated by varying the fixed‐effect coefficient over a grid from *B* = 0 to 0.5 in steps of 0.025. The target power of 80% was reached at approximately |*B*| = 0.375 (power = 80.0%, 95% CI [76.2%, 83.4%]). The observed group effect in the empirical analysis exceeded this threshold, indicating that the present design had sufficient sensitivity to detect the observed group difference in heart rate variability.

*Main Effect of Familiarity*

For HR, sensitivity for the main effect of familiarity was evaluated by varying the fixed-effect coefficient over a grid from *B* = 0 to 1.0 in steps of 0.05. The target power of 80% was reached at approximately |*B*| = 0.50 (power = 80.0%, 95% CI [76.2%, 83.4%]), indicating that the present design was sensitive to detect moderate familiarity-related effects on heart rate, whereas smaller effects would have been detected with substantially lower power.

For HRV, sensitivity for the main effect of familiarity was evaluated by varying the fixed-effect coefficient over a grid from *B* = 0 to 0.05 in steps of 0.005. The target power of 80% was reached at approximately |*B*| = 0.025 (power = 83.0%, 95% CI [79.4%, 86.2%]), indicating that the present design was sufficiently sensitive to detect small-to-moderate familiarity-related effects on heart rate variability.

*Higher-Order Interactions in Autonomic Outcomes*

To quantify sensitivity for detecting complex interaction effects in the physiological outcomes, we conducted simulation-based sensitivity analyses focusing on higher-order interactions that were central to the interpretation of the autonomic results.

For heart rate (HR), we evaluated sensitivity for the Group × Subject Gender × Partner Gender interaction by varying the fixed-effect coefficient of the three-way term over a grid from *B* = 0 to 13 in steps of 0.5. The target power of 80% was reached at approximately |*B|* = 11.0 (power = 83.0%, 95% CI [79.4%, 86.2%]). Compared to this threshold, the observed effect in the empirical analysis (*B* = −8.89) was smaller in magnitude, suggesting limited sensitivity for detecting three-way interaction effects of that size.

For heart rate variability (HRV), an analogous sensitivity analysis was conducted for the same three-way interaction by varying the fixed-effect coefficient over a grid from *B* = 0 to 1.0 in steps of 0.05. The target power of 80% was reached at approximately |*B|* = 0.50 (power = 79.0%, 95% CI [75.2%, 82.5%]). The observed three-way interaction estimate (*B* = 0.302, *p* = .442) fell below this threshold, indicating limited sensitivity to detect three-way HRV interaction effects of this magnitude under the present design.

## References

Bell, R. M., & McCaffrey, D. F. (2002). Bias reduction in standard errors for linear regression with multi-stage samples. *Survey Methodology*, *28*, 169–181.

Hartig, F. (2016). *DHARMa: Residual diagnostics for hierarchical (multi-level/mixed) regression models* [Computer software]. The R Foundation. https://cir.nii.ac.jp/crid/1360583646752175744

Huang, F., & Zhang, B. (2022). *CR2: Compute Cluster Robust Standard Errors with Degrees of Freedom Adjustments* (p. 0.2.1) [Computer software]. https://CRAN.R-project.org/package=CR2

Lüdecke, D., Ben-Shachar, M., Patil, I., Waggoner, P., & Makowski, D. (2021). performance: An R Package for Assessment, Comparison and Testing of Statistical Models. *Journal of Open Source Software*, *6*(60), 3139. https://doi.org/10.21105/joss.03139

Pustejovsky, J. E. (2016). *clubSandwich: Cluster-Robust (Sandwich) Variance Estimators with Small-Sample Corrections* (p. 0.6.1) [Computer software]. https://CRAN.R-project.org/package=clubSandwich
